# Surveillance for SARS‐CoV‐2 and its variants in wastewater of tertiary care hospitals correlates with increasing case burden and outbreaks

**DOI:** 10.1002/jmv.28442

**Published:** 2023-01-09

**Authors:** Nicole Acosta, Maria A. Bautista, Barbara J. Waddell, Kristine Du, Janine McCalder, Puja Pradhan, Navid Sedaghat, Chloe Papparis, Alexander Buchner Beaudet, Jianwei Chen, Jennifer Van Doorn, Kevin Xiang, Leslie Chan, Laura Vivas, Kashtin Low, Xuewen Lu, Jangwoo Lee, Paul Westlund, Thierry Chekouo, Xiaotian Dai, Jason Cabaj, Srijak Bhatnagar, Norma Ruecker, Gopal Achari, Rhonda G. Clark, Craig Pearce, Joe J. Harrison, Jon Meddings, Jenine Leal, Jennifer Ellison, Bayan Missaghi, Jamil N. Kanji, Oscar Larios, Elissa Rennert‐May, Joseph Kim, Steve E. Hrudey, Bonita E. Lee, Xiaoli Pang, Kevin Frankowski, John Conly, Casey R. J. Hubert, Michael D. Parkins

**Affiliations:** ^1^ Department of Microbiology, Immunology and Infectious Diseases University of Calgary Calgary Canada; ^2^ Department of Biological Sciences University of Calgary Calgary Canada; ^3^ Department of Mathematics and Statistics University of Calgary Calgary Canada; ^4^ C.E.C. Analytics Ltd. Calgary Canada; ^5^ Division of Biostatistics, School of Public Health University of Minnesota Minneapolis Minnesota USA; ^6^ Department of Community Health Sciences University of Calgary Calgary Canada; ^7^ Department of Medicine University of Calgary and Alberta Health Services Calgary Canada; ^8^ Provincial Population & Public Health Alberta Health Services Calgary Canada; ^9^ O'Brien Institute for Public Health University of Calgary Calgary Canada; ^10^ Faculty of Science and Technology Athabasca University Athabasca Alberta Canada; ^11^ Water Services, City of Calgary Calgary Canada; ^12^ Department of Civil Engineering University of Calgary Calgary Canada; ^13^ Infection Prevention and Control Alberta Health Services Calgary Canada; ^14^ Snyder Institute for Chronic Diseases University of Calgary and Alberta Health Services Calgary Canada; ^15^ Department of Laboratory Medicine and Pathology University of Alberta Edmonton Alberta Canada; ^16^ Alberta Precision Laboratories, Public Health Laboratory Alberta Health Services Edmonton Alberta Canada; ^17^ Department of Pathology and Laboratory Medicine University of Calgary and Alberta Health Services Calgary Canada; ^18^ Department of Analytical and Environmental Toxicology University of Alberta Edmonton Alberta Canada; ^19^ Department of Pediatrics University of Alberta Edmonton Alberta Canada; ^20^ Women & Children's Health Research Institute Edmonton Alberta Canada; ^21^ Li Ka Shing Institute of Virology University of Alberta Edmonton Alberta Canada; ^22^ Advancing Canadian Water Assets University of Calgary Calgary Canada

**Keywords:** COVID‐19, hospital‐acquired infection, prevalent, RT‐qPCR, variant of concern, wastewater‐based surveillance

## Abstract

Wastewater‐based SARS‐CoV‐2 surveillance enables unbiased and comprehensive monitoring of defined sewersheds. We performed real‐time monitoring of hospital wastewater that differentiated Delta and Omicron variants within total SARS‐CoV‐2‐RNA, enabling correlation to COVID‐19 cases from three tertiary‐care facilities with >2100 inpatient beds in Calgary, Canada. RNA was extracted from hospital wastewater between August/2021 and January/2022, and SARS‐CoV‐2 quantified using RT‐qPCR. Assays targeting R203M and R203K/G204R established the proportional abundance of Delta and Omicron, respectively. Total and variant‐specific SARS‐CoV‐2 in wastewater was compared to data for variant specific COVID‐19 hospitalizations, hospital‐acquired infections, and outbreaks. Ninety‐six percent (188/196) of wastewater samples were SARS‐CoV‐2 positive. Total SARS‐CoV‐2 RNA levels in wastewater increased in tandem with total prevalent cases (Delta plus Omicron). Variant‐specific assessments showed this increase to be mainly driven by Omicron. Hospital‐acquired cases of COVID‐19 were associated with large spikes in wastewater SARS‐CoV‐2 and levels were significantly increased during outbreaks relative to nonoutbreak periods for total SARS‐CoV2, Delta and Omicron. SARS‐CoV‐2 in hospital wastewater was significantly higher during the Omicron‐wave irrespective of outbreaks. Wastewater‐based monitoring of SARS‐CoV‐2 and its variants represents a novel tool for passive COVID‐19 infection surveillance, case identification, containment, and potentially to mitigate viral spread in hospitals.

## 1 INTRODUCTION

Successive waves of SARS‐CoV‐2 infection driven by different variants of concern (VOC) have been a prominent feature of the COVID‐19 pandemic. Many VOC exhibit reduced susceptibility to neutralizing antibodies and increased transmissibility, and manifest in variable disease severity.^1^, ^2^, ^3^ Variant emergence is a manifestation of frequent mutations within the SARS‐CoV‐2 genome,^4^ rapid transmission, and resultant selection pressures.

Wastewater‐based surveillance (WBS) has evolved to become a critical tool for population‐level COVID‐19 monitoring. This approach relies on detecting RNA from SARS‐CoV‐2 in wastewater shed in feces from presymptomatic, symptomatic and asymptomatic infected individuals.^5^, ^6^, ^7^, ^8^, ^9^ Diagnostic RT‐qPCR assays modified for wastewater have established strong correlations with clinically confirmed cases of COVID‐19 across a range of sewershed catchments (e.g., cities, neighborhoods, hospitals, public spaces, university campuses, individual buildings and even aircraft)^10^, ^11^, ^12^, ^13^, ^14^, ^15^, ^16^, ^17^, ^18^, ^19^, ^20^ and are increasingly used to guide public health policy. To understand the relative abundance/frequency of VOC in heterogeneous wastewater (i.e., potentially thousands of infected individuals contributing different VOC), several techniques has been described. Allele specific RT‐qPCR has allowed teams to monitor the emergence of variants in community sewage in Canada, Hong Kong, Israel, and United States.^21^, ^22^, ^23^, ^24^, ^25^, ^26^, ^27^

Herein, we used multiple RT‐qPCR assays to understand how total‐SARS‐CoV‐2 RNA and the differential abundance of VOC (Delta and Omicron) in wastewater correlated with the burden of COVID‐19 hospitalized individuals across three large tertiary care hospitals in Calgary, Canada. By categorizing cases as being community‐ or hospital‐acquired and identifying time periods corresponding to outbreaks, we were able to understand on a more granular scale the variation in SARS‐CoV‐2 VOC in wastewater systems as a function of fecal shedding during the disease timeline.

## 2 MATERIALS AND METHODS

### 2.1 Wastewater collection and sample processing

This research was approved by the Conjoint Health Research Ethics Board (REB‐20‐1252). Wastewater was collected thrice‐weekly from 08/09/2021 to 01/31/2022 at three tertiary care hospitals—spanning Calgary's successive Delta (mid‐August to November 2021) and Omicron (December–January 2022) waves. Hospital‐1 (NE Calgary, 517 inpatient beds) and Hospital‐2 (SW Calgary, 615 inpatient beds) were monitored by a single municipal access point each. Hospital‐3 (NW Calgary, ~1100 inpatient beds) required three separate access points encompassing separate sewers; Hospital‐3A included dedicated COVID‐19 care‐units and intensive care, and Hospital‐3B and Hospital‐3C represented the rest of the hospital. Wastewater collection and nucleic acid extraction is detailed in the Supplement.

### 2.2 RT‐qPCR and VOC RT‐qPCR analysis

**Figure 1:**
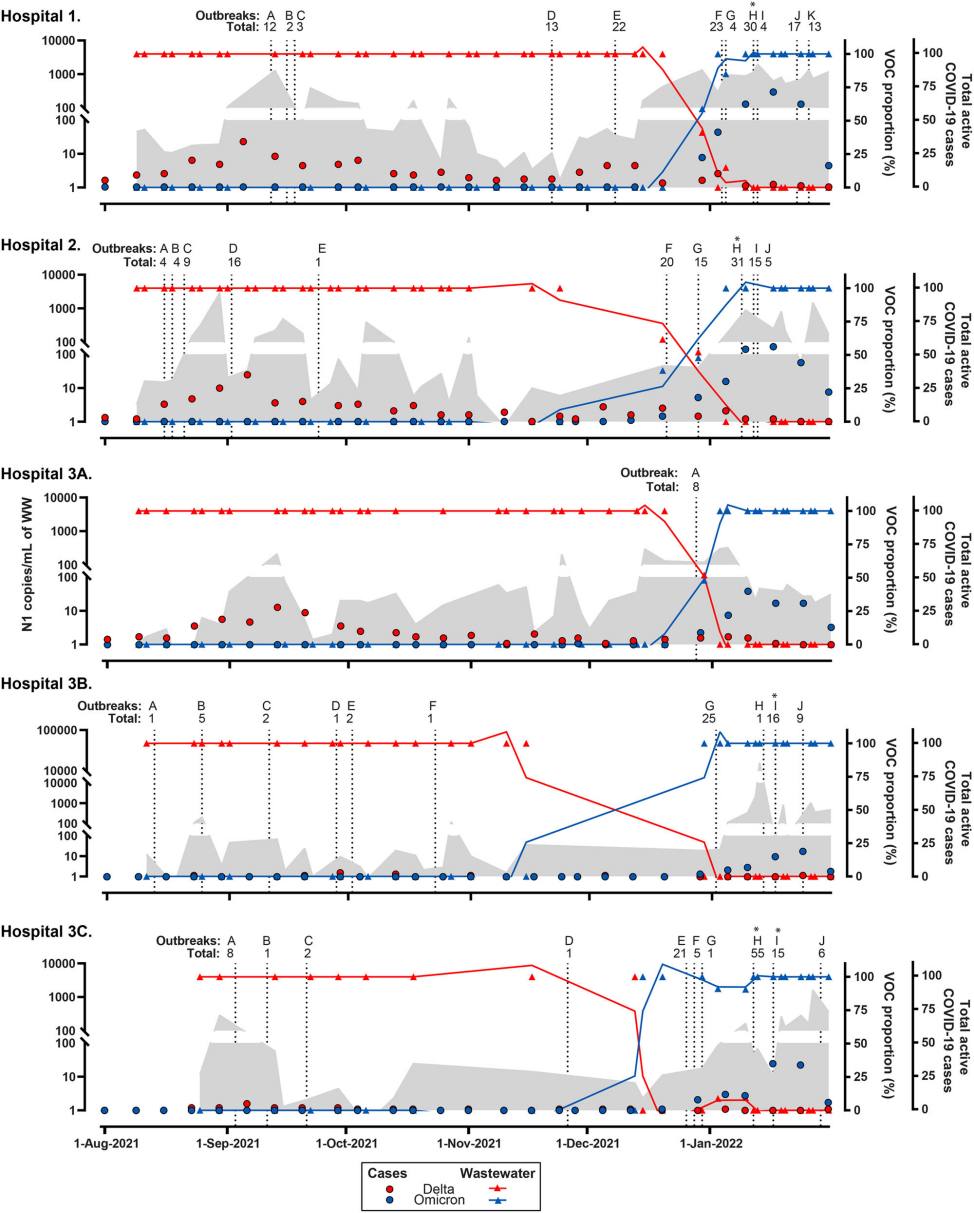
Daily census of COVID‐19 hospitalized individuals and SARS‐CoV‐2 RNA in hospital wastewater as a function of each variant of concern (VOC). Absolute concentration of SARS‐CoV‐2 RNA N1 signal (grey area), and the VOC proportion (%) of Delta (R203M mutation, red triangles) or Omicron (R203K/G204R mutation, blue triangles) in wastewater samples from five hospital locations: Hospital‐1, Hospital‐2, Hospital 3A, Hospital 3B; and Hospital 3C. The continuous blue and brown lines drawn through the triangle points are the lines of best fit plotted with the second order smoothing of the proportion of each mutation using GraphPad PRISM. N1 signal is presented in the left y‐axis and both VOC proportion (%) and smooth lines are presented in the first right y‐axis. Red and blue circles denote the weekly mean total number of prevalent cases for each VOC in the hospitals which is presented by the second right y‐axis. Vertical dash lines correspond to days where outbreaks were declared including the total number of individuals (i.e., patients plus health care workers) involved in each outbreak (Table 1). Asterisk denotes that for a specific outbreak more than one unit was involved. Please note that the N1 left Y‐axis scale is different for Hospital 3A. Since data in the left y‐axis is presented on a logarithmic 10 axis, it is not possible to plot nondetermined values (0)

The N1‐assay was used to quantify total SARS‐CoV‐2 RNA in wastewater. Samples were considered positive for N1 if the cycle threshold (Ct) was ≤40 cycles.^18^ We followed previously described protocols to estimate target gene abundances of an internal spiked control (i.e., Bovine Coronavirus) and a fecal biomarker (i.e., Pepper Mild Mottle Virus [PMMoV])^18^ (Figure 1S).

VOC detection was assessed with the N200 multiplex RT‐qPCR assay for the presence of N200‐universal, Delta (R203M) and Omicron (R203K/G204R) signals as previously described.^27^, ^28^ The N200 assay is a probe‐based multiplex assay that targets the region encoding amino acids 199‐202 within the nucleocapsid gene (N) which have been associated with variants of SARS‐CoV‐2.^28^ Serial dilutions of the TWIST AR‐S SARS‐CoV‐2 RNA control 14 and control 23 were run in triplicate on 96‐well PCR plates to produce standard curves used to quantify gene copies containing R203K/G204R and R203M mutations, respectively. RNA standards were prepared as single‐use aliquots. Standard curves for all RT‐qPCR assays were within an acceptable range for efficiencies and *R* ^2^ (Table 1S). All RT‐qPCRs were performed using a QuantStudio‐5 Real‐Time PCR System (Applied Biosystems). All experiments included no‐template controls. To estimate the VOC proportion (%), we first calculated the abundance (copies/ml) of each VOC from the copies per reaction using an established methodology.^18^ Then, we estimated the VOC proportion (%) of Delta (R203M mutation) or Omicron (R203K/G204R mutation) in RNA extracted from hospital wastewater by calculating the ratio of the abundance of a target mutation over the sum of the abundance of Omicron signal (R203K‐G204R assay) and Delta signal (R203M assay).^27^ All calculations for estimation of VOC proportions are described in the Supporting Information Materials. As the N200 assay does not discriminate between Alpha and Omicron variants, an assay targeting the nucleocapsid D3L mutation^22^ was performed to rule out the presence of the Alpha variant in the first and last samples that were positive for Omicron at each location.

### 2.3 COVID‐19 clinical case data from hospitals

The total COVID‐19 hospital census was documented daily for all locations. Daily COVID‐19 cases constituted the total community‐acquired (CA), hospital‐acquired (HA), and healthcare‐associated (HCA) cases and were adjudicated by trained Infection Prevention and Control practitioners of Alberta Health Services (AHS) using published definitions (Supporting Information Material).^29^ Cases were counted to a maximum of 14 days after admission (CA) or 14 days after their diagnosis (HCA/HA) during which time patients were managed with contact/droplet precautions, after which they were censored. All confirmed cases had variant testing for Delta or Omicron by specific mutation. If variant typing was not determined, results were reported as “unknown variant” (Table 2S). COVID‐19 outbreaks were defined as any unit with ≥1 confirmed HA case(s) and/or ≥2 confirmed COVID‐19 cases in health care workers (HCW) linked to a unit with no indication of acquired infection outside of workplace. Outbreak data, including dates, patients and HCW involved were collected from AHS (Table 3S).

### 2.4 Statistical analysis

SARS‐CoV‐2 copies/reaction were converted to copies/unit volume of wastewater as described previously.^18^ The sensitivity of the N1 and N200 universal assays was compared using McNemar test. Proportions of the Delta and Omicron variants within the total SARS‐CoV‐2 signal were calculated. Spearman correlation analyses were conducted to assess relationships between N1 and N200‐universal data, and total SARS‐CoV‐2 RNA level (N1 and N200) or VOC signal (R203K/G204R or R203M) against the daily total‐hospitalized COVID‐19 (i.e., CA, HA, and HCA) and HA cases. To compensate for gaps owing to wastewater being sampled thrice weekly relative to daily hospital data, HA cases occurring ± 2 days of wastewater collection were compared. Cross‐correlation function (CCF) analysis was performed to determine time‐lagged relationships between weekly average wastewater SARS‐CoV‐2 RNA‐level and weekly prevalent cases. Wastewater data and hospital‐case data were aggregated and analyzed by week for CCF analyses. A 95% confidence level was computed for the cross‐correlation values. To determine if differences in total SARS‐CoV‐2 RNA in wastewater occurred with outbreaks, wastewater SARS‐CoV‐2 N1 levels were compared during declared outbreaks and nonoutbreak periods using Mann–Whitney *U* test. Statistical analyses were conducted in GraphPad Prism‐8 software and in R (V4.0.4) using the forecast‐package.

## 3 RESULTS

### 3.1 Hospital wastewater SARS‐CoV‐2 RNA through the Delta and Omicron waves of COVID‐19

A total of 196 wastewater samples were collected from three tertiary‐care hospitals during Alberta's “fourth (Delta; mid‐August to November 2021) and fifth (Omicron; January 2022)‐waves” of COVID‐19. Ninety‐six percent of the samples were positive for SARS‐CoV‐2 using the N1‐assay. The N200 was less sensitive at 87.2% (*p* < 0.0001, McNemar's test). N1 copies/ml correlated with N200 across all samples (*r* = 0.91, *p* < 0.001).

The percentage of Delta and Omicron signal in hospital wastewater is presented in Figure 1. Total SARS‐CoV‐2 RNA was low (greyed area) in August and rapidly increased during Alberta's Delta wave in September. The SARS‐CoV‐2 RNA in wastewater was 100% Delta from mid‐August until early December (Figure 1, red lines/triangles). The proportion of Delta declined in mid‐December 2021 and Omicron emerged in all locations, accompanied by an increase in total SARS‐CoV‐2 wastewater RNA (Figure 1, blue lines/triangles). During mid‐December 2021 and early January 2022, there was greater discrimination of relative proportions (Delta to Omicron) across all hospitals (e.g., 61:39, 52:48, 42:58, 15:85, and 10:90 [Delta:Omicron], Table 4S). No Alpha‐strain was identified in 10 (5.1%) of samples tested during the Omicron wave. The decline of Delta and increase in Omicron variants in wastewater was mirrored by the changing prevalence of hospitalized individuals and their variant designations. By early August 2021 an increase in Delta hospitalized COVID‐19 individuals was observed across all hospital locations (Figure 1, red circles)—which peaked in September 2021. The first hospitalized Omicron case occurred on November 30th, 2021 (Figure 1, blue circles) and by January 27th only Omicron remained. All raw data for the gene abundance of targets analyzed and the percentage of Delta and Omicron signal are described in Table 4S.

### 3.2 SARS‐CoV‐2 RNA in wastewater correlates with the number of COVID‐19 hospitalized individuals

**Figure 2:**
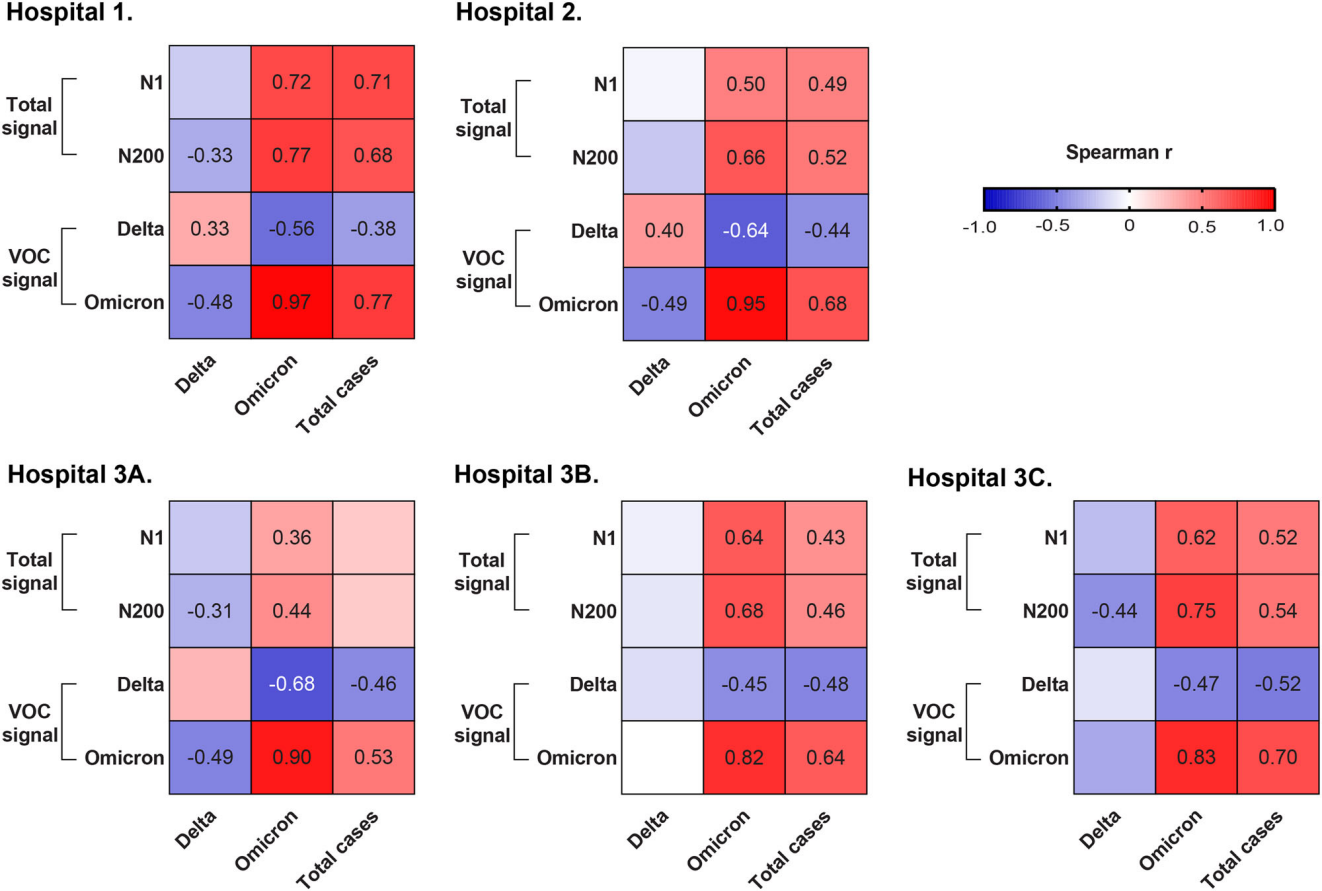
Association between total active COVID‐19 cases and wastewater SARS‐CoV‐2 RNA from hospitals. Heatmap of the Spearman analysis between daily cases (measured as Delta‐specifically, omicron‐specifically or total cases) and wastewater signal obtained with either the N1 assay or N200 assay or VOCs specific assays (i.e., R203M [Delta] or R203K/G204R [Omicron]) from monitored sites: Hospital 1, Hospital 2, Hospital 3A, Hospital 3B; and Hospital 3C. Spearman *r* value is only shown for those analysis when *p* < 0.05. VOC, variants of concern

**Figure 3:**
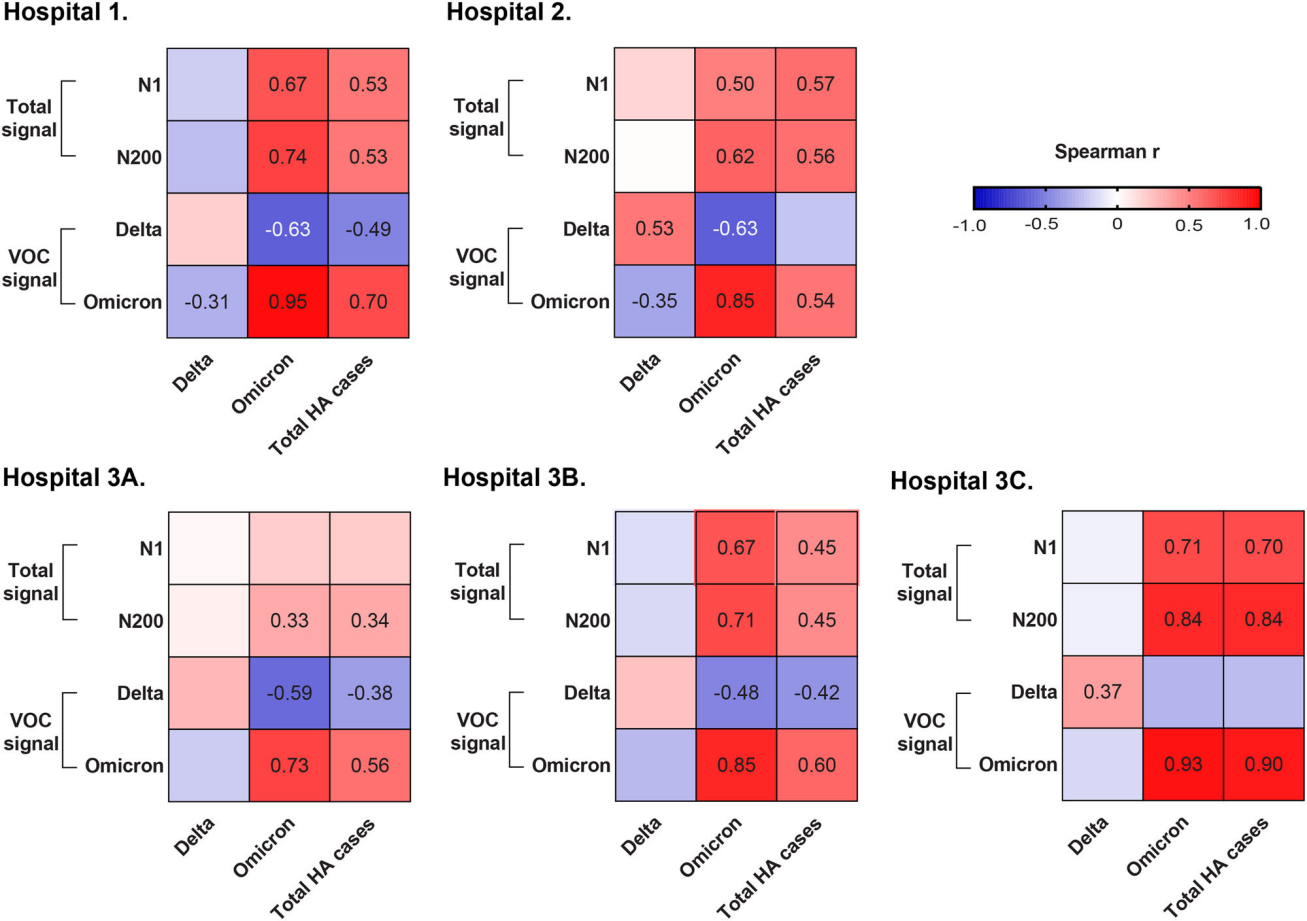
Association between hospital‐acquired (HA) COVID‐19 cases and wastewater SARS‐CoV‐2 signal from hospitals. Heatmap for the Spearman analysis between cases of COVID‐19 attributed to Delta, Omicron VOC and/or all active cases and wastewater signal obtained with either the N1 assay or N200 assay or VOCs specific assays (i.e., R203M [Delta] or R203K/G204R [Omicron]) from five hospital locations: Hospital 1, Hospital 2, Hospital 3A, Hospital 3B; and Hospital 3C. HA cases occurring ± 2 days were compared to wastewater signals. Spearman *r* value is only shown for those analysis when *p* < 0.05. VOC, variants of concern

**Figure 4:**
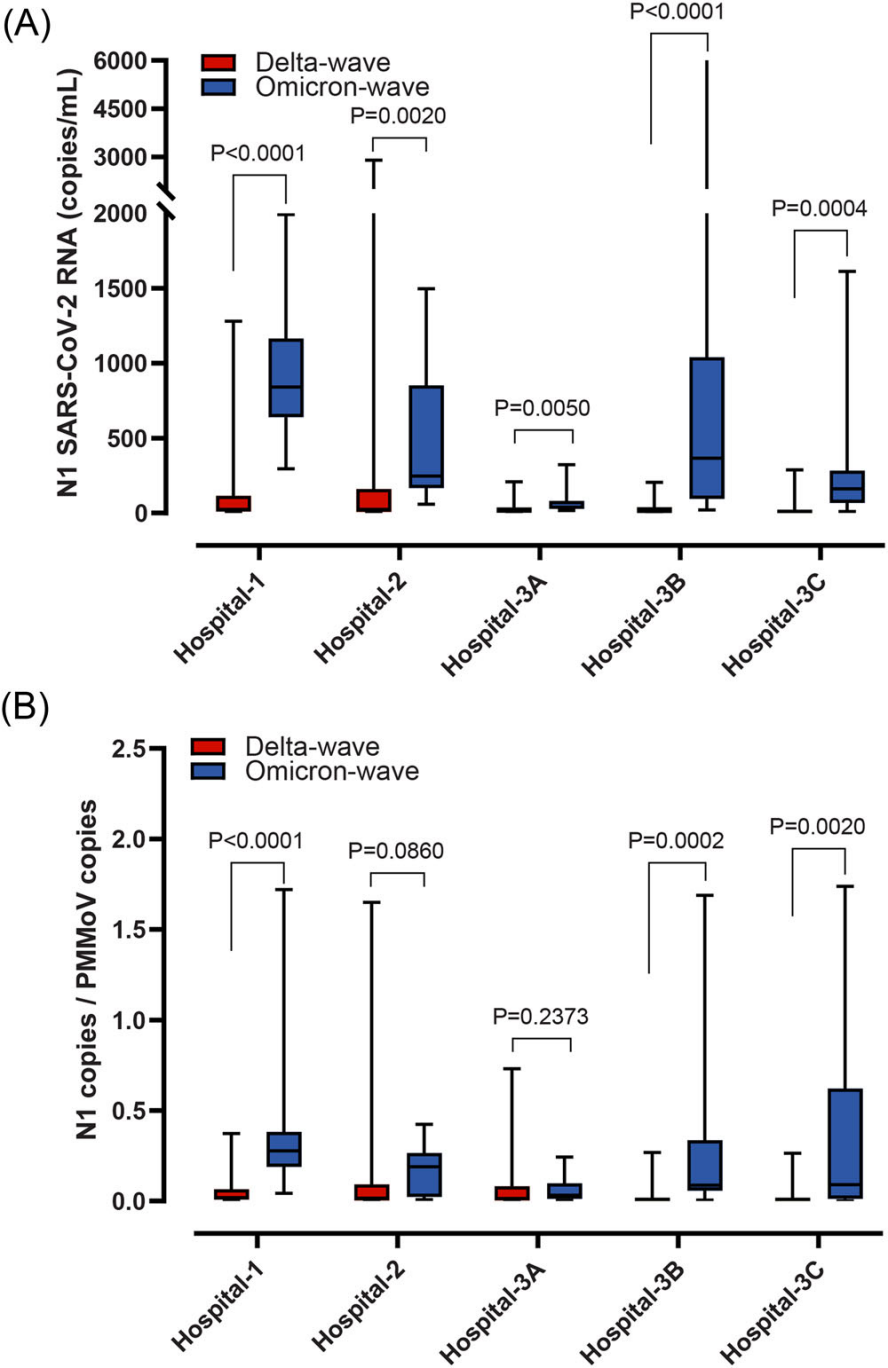
SARS‐CoV‐2 abundance in hospital wastewater as a function of VOC‐related waves. SARS‐CoV‐2 RNA data from the Delta‐wave (i.e., mid‐August to end of November 2021) were compared with samples collected during Omicron‐wave (i.e., January 2022). (A) N1 SARS‐CoV‐2 RNA signal (copies/ml). (B) N1 SARS‐CoV‐2 genomic copies normalized relative to genomic copies of the fecal biomarker PMMoV. Median and interquartile ranges are indicated as the middle, top, and bottom lines of each box. Ends of the whiskers mark the lowest and highest signal determined in each category for each hospital analyzed. Differences were determined using the Mann–Whitney *U* test. VOC, variants of concern

**Figure 5:**
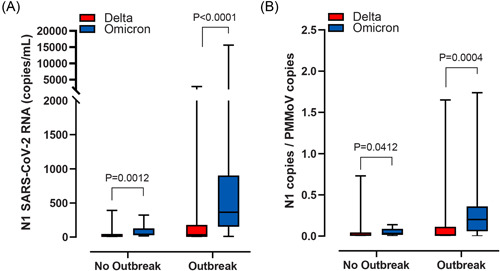
Aggregate abundance of SARS‐CoV‐2 wastewater signal during Delta or Omicron as a function of outbreak status. Aggregate SARS‐CoV‐2 RNA data from the Delta‐wave (i.e., mid‐August to end of November 2021) or Omicron‐wave (i.e., January 2022) were compared from samples collected during outbreak‐free periods or within 5 days of an outbreak being declared. (A) Combined N1 SARS‐CoV‐2 RNA signal (copies/ml) and (B) Combined N1 SARS‐CoV‐2 genomic copies normalized to genomic copies of the fecal biomarker PMMoV. Median and interquartile ranges are indicated as the middle, top, and bottom lines of each box. Ends of the whiskers mark the lowest and highest signal determined in each category for each hospital analyzed. Differences were determined using the Mann–Whitney *U* test. VOC, variants of concern

A positive correlation between the total number of hospitalized COVID‐19 cases and the total SARS‐CoV‐2 RNA level and specific variants in wastewater was observed across all hospital sites, with one exception [Hospital‐3A (Figure 2)]. The strongest correlation was observed at Hospital‐1 (*r* = 0.71, confidence interval [CI]: 0.53–0.83, *p* < 0.001 and *r* = 0.68, CI: 0.47–0.81, *p* < 0.001 for total SARS‐CoV‐2 RNA measured with N1 and N200 assays, respectively). A strong correlation between hospitalized COVID‐19 Omicron cases and level of Omicron SARS‐CoV‐2 RNA detected in wastewater was observed at all locations (median Spearman *r*: 0.9 (interquartile range: 0.83–0.95); Figure 2). A weaker correlation was found between the number of hospitalized Delta infected individuals and wastewater measured Delta at Hospitals‐1 and 2, respectively (*r* = 0.33, CI: 0.04–0.57, *p* = 0.025 and *r* = 0.40, CI: 0.09–0.64, *p* = 0.01) and no correlation observed at Hospital‐3 locations (Figure 2). Similar trends were observed when SARS‐CoV‐2 was normalized against the fecal biomarker PMMoV, albeit with lower Spearman *r*‐correlations (data not shown). Time series analysis of wastewater and cases was performed using CCF and detailed in the Supporting Information (Figures 2S–6S).

### 3.3 SARS‐CoV‐2 RNA in hospital wastewater correlates with hospital‐acquired COVID‐19 occurrence

Total SARS‐CoV‐2 in wastewater measured using either N1 or N200 assays correlated positively and significantly with the number of HA‐COVID‐19 cases at all hospital sites regardless of hospital COVID‐19 case burden with the exception of Hospital‐3A (Figure 3), where the mean N1 SARS‐CoV‐2 RNA level in wastewater was 2.2–11‐fold lower than other sites. The strongest correlation was observed at Hospital‐3C (*r* = 0.70, CI: 0.45–0.85, *p* < 0.001 and *r* = 0.9, CI: 0.67–0.92, *p* < 0.001 for N1 and N200, respectively) (Figure 3). We observed a moderate correlation between HA‐COVID‐19 cases typed as Delta and Delta‐RNA level in wastewater at Hospital‐2 (*r* = 0.53, CI: 0.25–0.73, *p* = 0.0005). A higher correlation between the Omicron RNA level in wastewater was found with the number of HA‐Omicron COVID‐19 cases at all locations where spearman *r*‐value ranged from 0.73 to 0.95 (Figure 3).

### 3.4 SARS‐CoV‐2 in wastewater increases in association with hospital outbreaks

Forty‐six outbreaks were declared during the study (Table 3S). Outbreaks coincided with an increase in the number of hospitalized COVID‐19 cases at each hospital and the burden of community COVID‐19 (https://covid-tracker.chi-csm.ca/), such that they clustered during two periods: mid‐August to the end of November 2021 and in January 2022 (Figure 1 and Table 3S). SARS‐CoV‐2 N1 was significantly increased in hospital wastewater during outbreaks relative to outbreak‐free periods for all locations (Table 1). The same trend was observed for all hospital locations except Hospital‐3A when wastewater SARS‐CoV‐2 was normalized for PMMoV. Similar results were obtained when total SARS‐CoV‐2 signal was evaluated using N200.

**Table 1:** Total SARS‐CoV‐2 RNA signal detection in hospital‐wastewater samples as a function of proximity to a declared outbreak

Hospital	Total SARS‐CoV‐2 RNA signal measured by	Measurement	Outbreak‐free periods vs. outbreaks [median (IQR)]	*p*‐value
1	N1	Copies/ml	45.5 (11.1–145) vs. 719 (76.9–1141)	<0.001
		Copies/copies PMMoV	2.6 × 10^−2^ (6.5 × 10^−3^–6 × 10^−2^) vs. 2.3 × 10^−1^ (4.1 × 10^−2^–3.7 × 10^−1^)	<0.001
	N200	Copies/ml	12.7 (3.1–20.3) vs. 347 (7.8–477)	0.001
		Copies/copies PMMoV	5.3 × 10^−3^ (1.6 × 10^−3^–1.3 × 10^−2^) vs. 8 × 10^−2^(7.7 × 10^−3^–1.8 × 10^−1^)	<0.001
2	N1	Copies/ml	9.9 (5.1–73.9) vs. 167 (39–354)	<0.001
		Copies/copies PMMoV	4.8 × 10^−3^ (1.7 × 10^−3^–6.6 × 10^−2^) vs. 3.7 × 10^−2^ (1.3 × 10^−2^–3 × 10^−1^)	0.005
	N200	Copies/ml	5.2 (0.7–18.5) vs. 36.6 (13.3–220)	<0.001
		Copies/copies PMMoV	3 × 10^−3^(2.6 × 10^−4^–1.4 × 10^−2^) vs. 1.5 × 10^−2^ (3.3 × 10^−3^–8.4 × 10^−2^)	0.007
3A	N1	Copies/ml	20.5 (3.9–47.1) vs. 211 (118–303)	0.033
		Copies/copies PMMoV	1.8 × 10^−2^ (4.9 × 10^−3^–7 × 10^−2^) vs. 1.2 × 10^−1^ (5.2 × 10^−3^–2.4 × 10^−1^)	0.723
	N200	Copies/ml	12.6 (2.7–35.8) vs. 204 (47.8–361)	0.052
		Copies/copies PMMoV	9.5 × 10^−3^ (2.1 × 10^−3^–3.5 × 10^−2^) vs. 1.5 × 10^−1^(2.1 × 10^−3^–2.9 × 10^−1^)	0.723
3B	N1	Copies/ml	10.3 (1.1–22.7) vs. 105 (4.8–447)	0.003
		Copies/copies PMMoV	4.5 × 10^−3^ (3.1 × 10^−4^–8.1 × 10^−3^) vs 6.1 × 10^−2^ (2.4 × 10^−3^–1.3 × 10^−1^)	0.008
	N200	Copies/ml	1.3 (0–7.4) vs. 34.7 (1.1–158)	0.006
		Copies/copies PMMoV	4.2 × 10^−^^4^ (0–3.7 × 10^−3^) vs. 2.1 × 10^−2^ (1.9 × 10^−4^–5.4 × 10^−2^)	0.014
3C	N1	Copies/ml	3.3 (0−13.5) vs. 77 (15.8−269)	0.003
		Copies/copies PMMoV	2.5 × 10^−3^ (0–1.1 × 10^−2^) vs. 5.2 × 10^−2^ (3.6 × 10^−3^–5.2 × 10^−1^)	0.003
	N200	Copies/ml	0.6 (0–5.3) vs. 91.6 (11.3–260)	<0.001
		Copies/copies PMMoV	2.3 × 10^−4^ (0–3.9 × 10^−3^) vs. 2.9 × 10^−2^ (2.2 × 10^−3^–5.2 × 10^−1^)	<0.001

Abbreviation: IQR, interquartile range.

The median SARS‐CoV‐2 N1 signal was higher during the Omicron wave than Delta wave across all hospital locations (Figure 4A). Similar results were observed when SARS‐CoV‐2 N1 signal was normalized for PMMoV except at sites Hospital‐2 and Hospital‐3A (Figure 4B). When we compared the SARS‐CoV‐2 signal attributed to Delta or Omicron during outbreaks vs outbreak‐free periods we observed a difference in VOC abundance for most sites, both raw and normalized (Figure S7A,B). When assessed in aggregate, we observed that total wastewater SARS‐CoV‐2 was higher during the Omicron‐wave relative to Delta, irrespective of outbreak occurrence, and when normalized for PMMoV (Figure 5A,B).

## 4 DISCUSSION

SARS‐CoV‐2 WBS conducted at wastewater treatment plants has proven a transformative tool in understanding the COVID‐19 pandemic. This strategy enables inclusive, objective and unbiased assessment of community COVID‐19 case burden.^30^ WBS is a leading indicator of cases,^10^, ^31^ hospitalizations^32^ and intensive care unit  (ICU) admissions.^33^ What was once a scientific curiosity is now routinely used globally to monitor COVID‐19 activity and to direct public health policy. However, key to untangling the aggregate wastewater signal (which can represent thousands of cases), is understanding clinical/case correlations on a more granular scale. Hospitals represent a unique model system in which to understand COVID‐19 wastewater dynamics. In hospitals, cases are concentrated, and reliable data exists for the population under study. Hospitals also represent a strategic priority as the implications of nosocomial transmission are particularly impactful as those with HA‐disease are likely to experience worse outcomes,^34^ and outbreaks disrupt healthcare delivery to a much broader population.^35^ Accordingly, tools that may act to identify and prevent HA infections is key.

We monitored the abundance of SARS‐CoV‐2 and its VOC in hospital wastewater during Calgary's fourth (Delta) and fifth (Omicron) COVID‐19 waves. We observed that by the beginning of December 2021, the Omicron variant had emerged in hospital wastewater and was already more abundant than Delta. By mid‐December, Omicron rapidly replaced Delta, and this correlated with lack of Delta‐associated hospital‐transmissions during a period of frequent hospital outbreaks. By the end of January 2022, there was no trace of Delta, and Omicron was the only variant detected in all hospital samples.

We found that the total COVID‐19 case population in each location was positively and significantly correlated with total SARS‐CoV‐2 in wastewater. Trends of wastewater VOC‐specific (Delta or Omicron) RNA followed closely and temporally the trends of daily total cases. Additionally, we found that an increase in the Omicron SARS‐CoV‐2 signal was correlated with a significant increase in Omicron‐related prevalent or incident COVID‐19 cases 2 weeks later.

Large spikes in hospital wastewater SARS‐CoV‐2 RNA were observed in the context of individual HA‐cases despite a background approximately 10%–15% of all patients in hospital recovering from COVID‐19 suggesting that fecal shedding peaks with initial symptom onset and drops rapidly thereafter.^18^ This suggests that WBS has significant potential for COVID‐19 infection surveillance in hospitals and other high‐risk institutions including long‐term care, and incarcerated populations where outbreaks abound. Indeed, a great effort has been directed to understand how outbreaks occur, and the use of whole genome sequencing to enable strain typing has ensured robust documentation of infection transmission chains.^36^ However, to this point a noninvasive early monitoring tool for detecting COVID‐19 outbreaks (and potentially other respiratory viral infections) has been lacking. With further refinement of this technology, WBS may fill this void.

Teasing out factors contributing towards total SARS‐CoV‐2 RNA signal represents a complex process. We observed a stronger correlation of wastewater measured SARS‐CoV‐2 with total hospitalized cases of COVID‐19 and specifically HA cases with Omicron VOC relative to Delta. This may be as SARS‐CoV‐2 levels were higher in hospital wastewater during the Omicron wave relative to Delta, even after controlling for outbreaks. Furthermore, protracted Delta virus shedding may reduce associations using our 14‐day definition of active‐disease.^37^ Finally, there is strong evidence that prolonged shedding occurs in those who are heavily immunosuppressed^38^ and in those with critical illness^39^ which were more common during Delta. Further studies that focus on specific hospital wards may shed more light on target sub‐populations.

While monitoring at wastewater treatment plants is a sustainable approach to monitoring COVID‐19 and the emergence of novel variants in communities, it is also important to monitor at a more granular scale (e.g., hospitals) since it can support the targeted protection of a population.^30^ For example, the Omicron wave in Alberta created a case burden that surpassed the capacity of the health care system to diagnose individual cases using RT‐qPCR assays—resulting in inaccurate case attainment data. However, resourced high‐risk sites such as hospitals continue to enable these direct comparisons to be made. Furthermore, variant monitoring of hospital wastewater could be used to guide empiric therapy for HA‐COVID‐19 as many therapies have variant‐variable activity.^40^ Additionally, the implementation of VOC WBS tools could benefit other health surveillance programs. For example, a study in Israel showed the importance of VOC monitoring tools on the understanding of immunity dynamics in a community.^41^

We observed correlations strongest at Hospital‐1 and ‐2 sites—facilities which used a single municipal access sampling point that captured each facility comprehensively. Hospital‐3 represented a more complex location, which required three separate monitoring locations (A, B, and C) to fully monitor the larger campus (>1100 beds). Because patients and staff frequently moved from one unit/building to another ascribing wastewater signal to any individual wastewater collection site was particularly challenging. For wastewater surveillance to be performed in hospital settings for other transmissible agents (i.e., antimicrobial resistant organisms, and other respiratory viruses)—it will be critical to understand patient movement across complex sites. While we attempted to control for potential differences in the amount of fecal matter in hospital wastewater (potentially more important in facility‐based studies than wastewater treatment plants) by normalizing against PMMoV during secondary analyses, we observed correlations that were actually lower. These observations are consistent with what has been published at a range of scales and indicates a great deal remains to be learnt from wastewater studies.^10^, ^42^, ^43^

Although this study adds in understanding VOC dynamics in complex wastewater systems there are limitations that warrant discussion. We were limited to thrice‐weekly sample collection due to reliance on municipal service partners and costs. Given the speed with which COVID‐19 spreads across high‐risk facilities, daily wastewater monitoring would be ideal to detect and mitigate further spread in real‐time and enable stronger comparisons with administrative data. Second, approximately 20% of COVID‐19 cases could not be attributed to either the Delta or Omicron variant (Table 2S), usually owing to low abundance of RNA in respiratory clinical specimens (i.e., RT‐qPCR with Ct >35), preventing these cases from being conclusively linked to wastewater variants. However, it is also likely that these individuals with low respiratory shedding also contributed lower SARS‐CoV‐2 RNA into sewage. Third, as WBS only detects signal from those individuals contributing to the sewage network we acknowledge that this technique fails to identify individuals who are not self‐toileting (i.e., immobile individuals dependent on continence aids, diapers, rectal tubes, etc. which are often disposed of via alternate routes). As a result, wastewater sampling may miss 10%–20% of hospitalized individuals including those in intensive care settings.^44^, ^45^ This was illustrated at Hospital‐3A where neither total hospitalized nor HA‐ COVID‐19 cases correlated with wastewater SARS‐CoV‐2. At the time, Hospital‐3A included 120 inpatient beds including 54 in the ICU where >85% of individuals were intubated and ventilated—and not contributing to the sewer system (evidenced by the lowest levels of SARS‐CoV‐2 and PMMoV). Finally, it is also important to highlight that because of the intrinsic high rate of mutations associated with the coronavirus genome, successive variants of SARS‐CoV‐2 are expected, and existing assays will be relevant for only short periods of time. In that light, research groups have already developed or adapted existing tools for tracking the introduction and spread of Omicron sub‐variants: BA.1, BA.2, BA.4, and BA.5.^24^, ^26^

Over 6 months we were able to ascribe SARS‐CoV‐2 in the wastewater of Calgary's three largest tertiary‐care hospitals to specific variants. Wastewater SARS‐CoV‐2 abundance correlated with increasing burden of individuals hospitalized with COVID‐19, acutely occurring HA‐disease and outbreaks. This study reveals the potential of WBS within hospitals for early detection, monitoring and containment of SARS‐CoV‐2 and its VOC incident infections.

## AUTHOR CONTRIBUTIONS

**Nicole Acosta**: formal analysis, investigation, writing ‐ original draft, writing ‐ review & editing. **Maria A. Bautista**: investigation, writing ‐ review & editing. **Barbara J. Waddell**: investigation, writing ‐ review & editing. **Kristine Du**: investigation. **Janine McCalder**: investigation, writing ‐ review & editing. **Puja Pradhan**: investigation. **Navid Sedaghat**: investigation. **Chloe Papparis**: investigation. **Alexander Buchner Beaudet**: investigation, writing ‐ review & editing. **Jianwei Chen**: writing ‐ review & editing. **Jennifer Van Doorn**: Investigation. **Kevin Xiang**: investigation. **Leslie Chan**: investigation. **Laura Vivas**: investigation, writing ‐ review & editing. **Kashtin Low**: investigation. **Xuewen Lu**: writing ‐ review & editing. **Jangwoo Lee**: investigation, writing ‐ review & editing. **Paul Westlund**: writing ‐ review & editing. **Thierry Chekouo**: formal analysis, writing ‐ review & editing. **Xiaotian Dai**: formal analysis, writing ‐ review & editing. **Jason Cabaj**: formal analysis, writing ‐ review & editing. **Srijak Bhatnagar**: writing ‐ review & editing. **Norma Ruecker**: conceptualization, writing ‐ review & editing. **Gopal Achari**: formal analysis, writing ‐ review & editing. **Rhonda G. Clark**: investigation, writing ‐ review & editing. **Craig Pearce**: formal analysis, writing ‐ review & editing. **Joe J. Harrison**: formal analysis, writing ‐ review & editing. **Jon Meddings**: formal analysis, writing ‐ review & editing. **Jenine Leal**: formal analysis, writing ‐ review & editing. **Jennifer Ellison**: formal analysis, writing ‐ review & editing. **Bayan Missaghi**: formal analysis, writing ‐ review & editing. **Jamil N. Kanji**: formal analysis, writing ‐ review & editing. **Oscar Larios**: formal analysis, writing ‐ review & editing. **Elissa Rennert‐May**: formal analysis, writing ‐ review & editing. **Joseph Kim**: formal analysis, writing ‐ review & editing. **Steve Hrudey**: formal analysis, writing ‐ review & editing. **Bonita E. Lee**: formal analysis, writing ‐ review & editing. **Xiaoli Pang**: formal analysis, writing ‐ review & editing. **Kevin Frankowski**: conceptualization, formal analysis, writing ‐ review & editing. **John Conly**: formal analysis, writing ‐ review & editing. **Casey R.J. Hubert**: conceptualization, formal analysis, funding acquisition, writing ‐ review & editing. **Michael D. Parkins**: conceptualization, formal analysis, funding acquisition, supervision, writing ‐ review & editing.

## CONFLICTS OF INTEREST

The authors declare no conflicts of interest.

## Supporting information

Supplementary information.

## Data Availability

The data that supports the findings of this study are available in the Supporting Information Material of this article.
