# Supplementary material for: Surveillance for SARS‐CoV‐2 and its variants in wastewater of tertiary care hospitals correlates with increasing case burden and outbreaks
Source: J Med Virol. 2023 Jan 9;95(2):e28442. doi: 10.1002/jmv.28442 (PMC9880705; doi:10.1002/jmv.28442)
Supplement: Supplementary file 1 — Supplementary information. [file JMV-95-0-s001.docx]

**SUPPLEMENTARY MATERIAL FOR:**

**Surveillance for SARS-CoV-2 and its variants in wastewater of tertiary care hospitals correlates with increasing case burden and outbreaks**

**SUPPLEMENTARY METHODS**

**Wastewater collection and sample processing**

Twenty-four-hour composite samples were collected using ISCO GLS autosamplers (Lincoln, Nebraska) programmed to collect 100 ml of wastewater every 15 mins, for a total of 96 pooled samples, following a previously described methodology ^1^. Wastewater samples were collected by City of Calgary staff and/or the university research team and shipped in coolers to the University of Calgary’s Advancing Canadian Water Assets (ACWA) laboratory. Samples in 10 L carboys were transported to the City of Calgary’s wastewater laboratory on ice where, after vigorous mixing, were sub-divided and stored at 4°C. Sample extraction and molecular analysis were performed at separate sites to prevent cross contamination. Extraction was performed as previously described using the Sewage, Salt, Silica, and SARS-CoV-2 (4S) protocol^1,2^. In brief, 40 ml of each sample was spiked with a Bovine coronavirus (BCoV) surrogate control and lysed via NaCl addition, followed by a filtration step, and the binding and subsequent elution of samples though a silica spin column. Extracted nucleic acids were transported on dry ice to the Health Sciences Centre for subsequent analysis.

**RT-qPCR and VOC RT-qPCR analysis**

To estimate the VOC proportion (%), we first calculated the abundance (copies/mL) of each VOC from the copies per reaction using Equation (1) that was previously described^1^.

Eq (1)

$$\text{VOC}=\left( \frac{\text{Copies per reaction * }\text{Volume of nucleic acids eluted }}{\text{Volume of nucleic acids assayed per reaction}} \right)\left( \frac{\text{1}}{\text{Volume of wastewater}} \right)$$

Then, we estimated the VOC proportion (%) of Delta (R203M mutation) or Omicron (R203K/G204R mutation) in RNA extracted from hospital wastewater by calculating the ratio of the abundance of a target mutation over the sum of the abundance of Omicron signal (R203K-G204R assay) and Delta signal (R203M assay) using Equation (2) that was previously described^3^.

$$VOC proportion \left( \% \right)=\left( \frac{\text{VOC1}\text{(copies per mL)}}{\text{VOC1}\text{(copies per mL)}\text{ + }\text{VOC2}\text{(copies per mL)}} \right)*100$$

Eq (2)

**COVID-19 Clinical Case Data from Hospitals**

Individuals in hospital with COVID-19 were categorized using standard definitions. Community-acquired (CA) cases were defined as all those individuals with a positive qPCR laboratory test for COVID-19 ≤14 days prior to admission, on the day of admission or any time up to and including day 6 of admission to any of the three tertiary-care hospitals and did not fulfill the criteria for Hospital-acquired (HA) or healthcare associated (HCA) infections. HA cases were defined as those individuals who were not admitted for COVID-19 but acquiring COVID-19 while in hospital (on or after the 7^th^ calendar day of admission) that were confirmed to have a positive clinical COVID-19 qPCR test representing a new infection and did not have evidence of community exposure before admission. HCA cases were defined as those individuals with positive laboratory COVID-19 qPCR testing within 14 days prior to admission, on the day of admission (calendar day 1) or anytime up to and including calendar day 6 (≤ 6 calendar days) to an inpatient location, and

COVID-19 infection was not acquired at any specific facility and individuals who were a direct transfer from a long-term care facility.

Outbreak details were identified and extracted using the Outbreak Tracker: COVID Outbreaks–Alberta Health Services (AHS) Tableau Server (albertahealthservices.ca). Cases were ascribed to specific VOC based on clinical testing. All confirmed cases underwent variant testing for Delta or Omicron by specific mutation using real-time RT-PCR ^4,5^.  Those in which variant RT-PCR did not provide a result were reported as “unresolved variant,” with no further testing carried out.  This most commonly occurs because the Cq of the positive result is >35 cycles.  SARS-CoV-2 genomic sequencing was conducted on positive specimens with a Cq≤30 and meeting any of the following criteria: patients involved in outbreaks, hospitalized/emergency room cases, inbound international travelers, and healthcare workers.  Sequencing was limited to these patient types because of increased positive results during a SARS-CoV-2 wave and strain on laboratory human resources.

**SUPPLEMENTARY RESULTS**

**Quantification of BCoV and PMMoV**

To determine reliability of nucleic acid detection in processed samples, Bovine Corona Virus (BCoV) was used as an internal control for sample processing and RNA purification. Sample recovery of BCoV was similar in wastewater among all hospital sites except for some samples from Hospital-3 locations A and B (Supplemental Figure 1S). The identified median number of spiked BCoV copies per ml of wastewater samples was the following: Hospital-1, 1.7 × 10^6^ (IQR, 9.8 × 10^5^– 3.2 × 10^6^); Hospital-2, 2.2 × 10^6^ (IQR, 1.1 × 10^6^– 4 × 10^6^); Hospital-3A, 9.6 × 10^5^ (IQR, 6.3 × 10^5^– 1.4 × 10^6^); Hospital-3B, 9.4 × 10^5^ (IQR, 4.9 × 10^5^– 1.6 × 10^6^); and Hospital3-C, 1.3 × 10^6^ (IQR, 7.3 × 10^5^– 2.4 × 10^6^). The wastewater fecal biomarker Pepper Mild Mottle Virus (PMMoV) concentration varied from 59.3 to 58,397 genomic copies/ml across all hospital sites (Supplemental Figure 1S). The median number of PMMoV copies per ml of wastewater samples was: Hospital-1, 2.1 × 10^3^ (IQR, 9.7 × 10^2^– 4.9 × 10^3^); Hospital-2, 1.9 × 10^3^ (IQR, 9.5 × 10^2^– 7.3 × 10^3^); Hospital-3A, 1.3 × 10^3^ (IQR, 2.7 × 10^2^– 3.1 × 10^3^); Hospital-3B, 2.7 × 10^3^ (IQR, 1.2 × 10^3^– 8.3 × 10^3^); and Hospital3-C, 1.3 × 10^3^ (IQR, 6 × 10^2^– 5.5 × 10^3^). PMMoV did differ across hospital sites (P=0.03, Kruskal-Wallis test). All reactions included no template controls which were negative for amplified product.

**Clinical Case Data**

Hospital admissions of COVID-19 coincided with increased COVID-19 in the community as indicated by city-wide SARS-CoV-2 RNA wastewater values (data not shown; CSM COVID tracker; <https://covid>-tracker.chi-csm.ca).

During the study 20.32% of COVID-19 clinical diagnoses could not be attributed to any individual VOC, and this did not differ during Delta and Omicron waves.

All clinical cases in hospital underwent strain identification using established methods. Twenty-three percent of COVID-19 clinical diagnoses were classified as unknown variant (generally when Cq values were >35), and this percentage did not differ during Delta and Omicron waves (Supplementary Table 1S).

**SARS-CoV-2 RNA in wastewater correlates with the number of COVID-19 hospitalized individuals**

Using a CCF analysis, the highest cross correlations values between the total level of SARS-CoV-2 wastewater N1 and total hospitalized COVID-19 cases occurred between -3 and 1 lags (weeks). The highest cross-correlation values were 0.62, 0.56, 0.56, 0.83 and 0.97 for Hospital-1, Hospital-2, Hospital-3A, Hospital-3B and Hospital-3C; respectively (Supplementary Figures 2S to 6S, panel A). Similar results were observed when total SARS-CoV-2 was measured with N200 (Supplementary Figures 2S to 6S, panel B). For VOC-specific assessments, a CCF analysis for the wastewater Delta data showed a significant correlation at lag -1 for Hospital-2 and Hospital-3C only (Cross-correlation values 0.73 and 0.80, respectively, *P*<0.05) (Supplementary Figures 2S to 6S, panel C). On the other hand, CCF analysis for the wastewater Omicron data showed a positive association at all hospital sites, where the highest cross-correlation was between lags -2 and 1 (CCF value range between 0.75-1, *P*<0.05) (Supplementary Figures 2S to 6S, panel D).

**SARS-CoV-2 RNA in hospital wastewater correlates with hospital-acquired COVID-19 occurrence**

CCF analysis (Supplementary Figures 2-6) showed that the maximum cross-correlation values for the analysis between wastewater total SARS-CoV-2 RNA levels measured by either N1 or N200 and HA cases were at the lag (-1) for Hospital-3 location B (0.96 and 0.96, N1 and N200 respectively, *P*<0.05). Wastewater SARS-CoV-2 RNA concentration had a positive trend and led HA-cases by 1 week (Supplementary Figure 5E and 5F). Similar patterns were observed for Hospital-1, 2 and 3-location-A, where there were significant correlations between weekly total SARS-CoV-2 RNA levels in wastewater and HA-COVID-19 cases. The number of HA COVID-19 cases was positively correlated with the increase of SARS-CoV-2 signal 1-2 weeks before (Supplementary Figures 2-4, panels E and F; *P*<0.05). When we computed the CCF between the wastewater Delta and Omicron SARS-CoV-2 RNA signals and VOC-specific HA cases, at different lags, we observed a weak or no cross-correlation for wastewater Delta RNA levels and HA-cases (Supplementary Figures 2-6, panel G). However, cross-correlation in data series for Omicron showed a strong correlation between the number of weekly HA-Omicron COVID-19 cases and wastewater Omicron RNA between lag -2 to 1 for all hospital locations (Supplementary Figures 2-6, panel H).

**SUPPLEMENTARY TABLES**

**Table 1S.** RT-qPCR assays performance.

| Target | Y-intercept  (Median (IQR)) | Slope (Median (IQR)) | Efficiency (Median (IQR)) | R^2^  (Median (IQR)) |
| --- | --- | --- | --- | --- |
| N1 | 39.6 (39 – 40.2) | -3.1(-3.3 – -3) | 109.8(100.2 – 118.1) | 1 (1 – 1) |
| R203M | 41(40.5 – 41.5) | -3.5(-3.5 – -3.4) | 92.4(91.8 – 95.1) | 1 (1 – 1) |
| R203K-G204R | 42(41.5 – 42) | -3.7(-3.7 – -3.5) | 88 (87– 92.2) | 1 (1 – 1) |
| N200 universal | 39.3(39– 39.6) | -3.7(-3.7 – -3.6) | 87(86 – 91.2) | 1 (1 – 1) |
| BCoV | 40.4(38.5 – 43) | -3.4(-3.6 – -3.1) | 98.6 (88.4 – 109.6) | 1 (1 – 1) |
| PMMoV | 43.2(41 – 45.6) | -3.3(-3.6 – -3.1) | 101.7(89.5 – 112.3) | 1 (1 – 1) |

**Table 2S.** Weekly number of community-acquired (CA) and hospital-acquired (HA) cases that were recorded as status “unknown variant” (No healthcare associated (HCA) case was identified as unknown variant)

| Calendar week number* | Hospital | | | | | | | | | |
| --- | --- | --- | --- | --- | --- | --- | --- | --- | --- | --- |
|  | **1** | | **2** | | **3A** | | **3B** | | **3C** | |
|  | **CA** | **HA** | **CA** | **HA** | **CA** | **HA** | **CA** | **HA** | **CA** | **HA** |
| 32 | 0(0%) | 0(0%) | 1(25%) | 0(0%) | 0(0%) | 0(0%) | 0(0%) | 0(0%) | 0(0%) | 0(0%) |
| 33 | 1(10%) | 0(0%) | 0(0%) | 0(0%) | 1(12.5%) | 0(0%) | 0(0%) | 0(0%) | 1(100%) | 0(0%) |
| 34 | 0(0%) | 0(0%) | 0(0%) | 0(0%) | 0(0%) | 0(0%) | 0(0%) | 1(100%) | 0(0%) | 0(0%) |
| 35 | 2(9.1%) | 0(0%) | 0(0%) | 0(0%) | 1(6.7%) | 0(0%) | 0(0%) | 0(0%) | 0(0%) | 0(0%) |
| 36 | 1(5.6%) | 0(0%) | 0(0%) | 0(0%) | 1(5.9%) | 0(0%) | 0(0%) | 0(0%) | 2(50%) | 0(0%) |
| 37 | 2(5.6%) | 0(0%) | 2(7.7%) | 0(0%) | 1(5.6%) | 0(0%) | 0(0%) | 0(0%) | 0(0%) | 0(0%) |
| 38 | 8(28.6%) | 0(0%) | 5(27.8%) | 0(0%) | 5(15.2%) | 0(0%) | 0(0%) | 1(100%) | 1(50%) | 0(0%) |
| 39 | 6(27.3%) | 1(100%) | 6(28.6%) | 1(100%) | 5(17.2%) | 0(0%) | 0(0%) | 0(0%) | 3(75%) | 0(0%) |
| 40 | 6(26.1%) | 0(0%) | 4(25%) | 0(0%) | 7(33.3%) | 0(0%) | 1(50%) | 0(0%) | 0(0%) | 0(0%) |
| 41 | 11(35.5%) | 0(0%) | 9(40.9%) | 0(0%) | 4(28.6%) | 0(0%) | 0(0%) | 0(0%) | 0(0%) | 0(0%) |
| 42 | 6(37.5%) | 0(0%) | 5(38.5%) | 0(0%) | 5(35.7%) | 0(0%) | 0(0%) | 0(0%) | 1(50%) | 0(0%) |
| 43 | 2(18.2%) | 0(0%) | 2(14.3%) | 0(0%) | 5(45.5%) | 0(0%) | 1(100%) | 0(0%) | 0(0%) | 0(0%) |
| 44 | 6(35.3%) | 0(0%) | 3(42.9%) | 0(0%) | 1(16.7%) | 0(0%) | 0(0%) | 0(0%) | 0(0%) | 0(0%) |
| 45 | 2(22.2%) | 0(0%) | 0(0%) | 0(0%) | 3(30%) | 0(0%) | 0(0%) | 0(0%) | 0(0%) | 0(0%) |
| 46 | 7(58.3%) | 0(0%) | 2(22.2%) | 0(0%) | 2(66.7%) | 0(0%) | 0(0%) | 0(0%) | 1(100%) | 0(0%) |
| 47 | 3(33.3%) | 0(0%) | 1(100%) | 0(0%) | 2(20%) | 0(0%) | 1(100%) | 0(0%) | 0(0%) | 0(0%) |
| 48 | 1(20%) | 0(0%) | 3(42.9%) | 0(0%) | 0(0%) | 0(0%) | 0(0%) | 0(0%) | 1(50%) | 0(0%) |
| 49 | 0(0%) | 0(0%) | 1(33.3%) | 0(0%) | 3(33.3%) | 0(0%) | 0(0%) | 0(0%) | 1(100%) | 0(0%) |
| 50 | 1(11.1%) | 1(11.1%) | 0(0%) | 0(0%) | 0(0%) | 0(0%) | 0(0%) | 0(0%) | 0(0%) | 0(0%) |
| 51 | 2(18.2%) | 0(0%) | 0(0%) | 0(0%) | 0(0%) | 0(0%) | 0(0%) | 0(0%) | 2(66.7%) | 0(0%) |
| 52 | 1(14.3%) | 0(0%) | 2(14.3%) | 0(0%) | 2(33.3%) | 0(0%) | 0(0%) | 0(0%) | 1(50%) | 1(100%) |
| 53 | 2(6.9%) | 0(0%) | 4(15.4%) | 0(0%) | 3(17.6%) | 0(0%) | 1(33.3%) | 0(0%) | 2(28.6%) | 0(0%) |
| 2 | 7(12.9%) | 0(0%) | 2(5.3%) | 0(0%) | 10(26.3%) | 0(0%) | 1(16.7%) | 0(0%) | 2(22.2%) | 0(0%) |
| 3 | 14(19.7%) | 0(0%) | 8(17.4%) | 2(10%) | 4(8.5%) | 0(0%) | 2(28.6%) | 0(0%) | 2(22.2%) | 1(20%) |
| 4 | 25(30.8%) | 3(15%) | 11(18.0%) | 4(33.3%) | 14(32.6%) | 0(0%) | 3(33.3%) | 0(0%) | 8(29.6%) | 0(0%) |
| 5 | 20(28.1%) | 2(14.3%) | 12(25.5%) | 2(16.6%) | 11(27.5%) | 0(0%) | 5(23.8%) | 0(0%) | 4(17.4%) | 3(16.7%) |
| 6 | 5(27.8%) | 0(0%) | 7(38.9%) | 0(0%) | 3(30%) | 1(100%) | 0(0%) | 1(50%) | 1(50%) | 0(0%) |

Data is presented as n (%). CA: community-acquired, HA: hospital-acquired. *week 32 to 52: 2021 and week 2 to 6: 2022

**Table 3S**. Declared outbreaks occurring in three tertiary-care hospitals during Alberta’s “fourth and fifth-wave” of COVID-19.

| Hospital | Outbreak ID | Date (First-Last case) | Number of Patients | Number of HCW | Total |
| --- | --- | --- | --- | --- | --- |
| 1 | A | Sep 12 – Oct 13, 2021 | 6 | 6 | 12 |
|  | B | Sep 16 – Sep 17, 2021 | 2 | 0 | 2 |
|  | C | Sep 18 – Sep 24, 2021 | 1 | 2 | 3 |
|  | D | Nov 22 – Dec 7, 2021 | 10 | 3 | 13 |
|  | E | Dec 8, 2021 – Jan 3, 2022 | 13 | 9 | 22 |
|  | F | Jan 4 – Jan 25, 2022 | 11 | 12 | 23 |
|  | G | Jan 5 – Jan 8, 2022 | 3 | 1 | 4 |
|  | H-1* | Jan 12 – Jan 31, 2022 | 16 | 4 | 20 |
|  | H-2* | Jan 12 – Jan 28, 2022 | 4 | 6 | 10 |
|  | I | Jan 13 – Jan 18, 2022 | 2 | 2 | 4 |
|  | J | Jan 23 – Feb 9, 2022 | 10 | 7 | 17 |
|  | k | Jan 26 – Feb 6, 2022 | 7 | 6 | 13 |
| 2 | A | Aug 16 – Aug 20, 2021 | 2 | 2 | 4 |
|  | B | Aug 18 – Aug 23, 2021 | 2 | 2 | 4 |
|  | C | Aug 21 – Sep 6, 2021 | 7 | 2 | 9 |
|  | D | Sep 2 – Sep 18, 2021 | 11 | 5 | 16 |
|  | E | Sep 24 – Sep 24, 2021 | 1 | 0 | 1 |
|  | F | Dec 21 – Jan 25, 2022 | 7 | 13 | 20 |
|  | G | Dec 29 – Feb 3, 2022 | 8 | 7 | 15 |
|  | H-1* | Jan 9 – Jan 12, 2022 | 2 | 4 | 6 |
|  | H-2* | Jan 9 – Feb 7, 2022 | 18 | 7 | 25 |
|  | I | Jan 12 – Jan 23, 2022 | 11 | 4 | 15 |
|  | J | Jan 13 – Jan 22, 2022 | 4 | 1 | 5 |
| 3A | A | Dec 28 – Jan 15, 2022 | 3 | 5 | 8 |
| 3B | A | Aug 13 – Aug 13, 2021 | 1 | 0 | 1 |
|  | B | Aug 25 – Sep 4, 2021 | 4 | 1 | 5 |
|  | C | Sep 11 – Sep 17, 2021 | 1 | 1 | 2 |
|  | D | Sep 28 – Sep 28, 2021 | 1 | 0 | 1 |
|  | E | Oct 2 – Oct 4, 2021 | 2 | 0 | 2 |
|  | F | Oct 23 – Oct 23, 2022 | 1 | 0 | 1 |
|  | G | Jan 2 – Feb 20, 2022 | 18 | 7 | 25 |
|  | H | Jan 14 – Jan 14, 2022 | 1 | 0 | 1 |
|  | I-1* | Jan 17– Jan 18, 2022 | 0 | 2 | 2 |
|  | I-2* | Jan 17– Jan 26, 2022 | 8 | 6 | 14 |
|  | J | Jan 24 – Feb 8, 2022 | 7 | 2 | 9 |
| 3C | A | Sep 3 – Sep 13, 2021 | 5 | 3 | 8 |
|  | B | Sep 11 – Sep 11, 2021 | 1 | 0 | 1 |
|  | C | Sep 21 – Sep 23, 2021 | 1 | 1 | 2 |
|  | D | Nov 26 – Nov 26, 2021 | 1 | 0 | 1 |
|  | E | Dec 26 – Jan 22, 2022 | 9 | 12 | 21 |
|  | F | Dec 28 – Jan 27, 2022 | 3 | 2 | 5 |
|  | G | Dec 30 – Dec 30, 2022 | 1 | 0 | 1 |
|  | H-1* | Jan 12 – Feb 6, 2022 | 21 | 14 | 35 |
|  | H-2* | Jan 12 – Feb 12, 2022 | 11 | 9 | 20 |
|  | I-1* | Jan 17 – Jan 25, 2022 | 4 | 3 | 7 |
|  | I-2* | Jan 17 – Feb 3, 2022 | 7 | 1 | 8 |
|  | J | Jan 29 – Feb 2, 2022 | 3 | 3 | 6 |

HCW: health care workers

**Table 4S**. Raw data of the calculated copies per mL for N1, N200 universal, Delta and Omicron; and VOC proportion (%).

| Location | Collection date | N1 (copies/mL) | N200 Universal (copies/mL) | Delta (R203M) (copies/mL) | Omicron (R203K/G204R) (copies/mL) | Delta (R203M) (%) | Omicron (R203K/ G204R) (%) |
| --- | --- | --- | --- | --- | --- | --- | --- |
| Hospital 1 | 2021-08-09 | 45.38 | 19.17 | 32.31 | 0.00 | 100.00 | 0.00 |
| Hospital 2 | 2021-08-09 | 3.26 | 3.56 | 5.09 | 0.00 | 100.00 | 0.00 |
| Hospital 3A | 2021-08-09 | 1.18 | 1.36 | 1.84 | 0.00 | 100.00 | 0.00 |
| Hospital 3B | 2021-08-09 | 0.00 | 0.00 | 0.00 | 0.00 | 0.00 | 0.00 |
| Hospital 3C | 2021-08-09 | 0.00 | 0.00 | 0.00 | 0.00 | 0.00 | 0.00 |
| Hospital 1 | 2021-08-11 | 52.20 | 18.15 | 27.23 | 0.00 | 100.00 | 0.00 |
| Hospital 2 | 2021-08-11 | 16.14 | 16.32 | 24.41 | 0.00 | 100.00 | 0.00 |
| Hospital 3A | 2021-08-11 | 1.72 | 5.29 | 7.75 | 0.00 | 100.00 | 0.00 |
| Hospital 3B | 2021-08-11 | 12.43 | 6.62 | 9.56 | 0.00 | 100.00 | 0.00 |
| Hospital 3C | 2021-08-11 | 0.00 | 0.00 | 0.00 | 0.00 | 0.00 | 0.00 |
| Hospital 1 | 2021-08-16 | 11.31 | 4.30 | 6.12 | 0.00 | 100.00 | 0.00 |
| Hospital 2 | 2021-08-16 | 14.87 | 8.20 | 12.95 | 0.00 | 100.00 | 0.00 |
| Hospital 3A | 2021-08-16 | 2.88 | 4.48 | 6.68 | 0.00 | 100.00 | 0.00 |
| Hospital 3B | 2021-08-16 | 0.00 | 0.00 | 0.00 | 0.00 | 0.00 | 0.00 |
| Hospital 3C | 2021-08-16 | 0.00 | 0.00 | 0.00 | 0.00 | 0.00 | 0.00 |
| Hospital 1 | 2021-08-18 | 10.77 | 4.76 | 6.45 | 0.00 | 100.00 | 0.00 |
| Hospital 2 | 2021-08-18 | 16.96 | 14.49 | 22.03 | 0.00 | 100.00 | 0.00 |
| Hospital 3A | 2021-08-18 | 0.58 | 0.00 | 0.00 | 0.00 | 0.00 | 0.00 |
| Hospital 3B | 2021-08-18 | 0.37 | 0.00 | 0.00 | 0.00 | 0.00 | 0.00 |
| Hospital 3C | 2021-08-18 | 0.00 | 0.00 | 0.00 | 0.00 | 0.00 | 0.00 |
| Hospital 1 | 2021-08-23 | 17.57 | 12.84 | 18.02 | 0.00 | 100.00 | 0.00 |
| Hospital 2 | 2021-08-23 | 152.60 | 36.44 | 55.18 | 0.00 | 100.00 | 0.00 |
| Hospital 3A | 2021-08-23 | 0.94 | 1.12 | 1.47 | 0.00 | 100.00 | 0.00 |
| Hospital 3B | 2021-08-23 | 108.02 | 11.96 | 17.78 | 0.00 | 100.00 | 0.00 |
| Hospital 3C | 2021-08-23 | 0.00 | 0.00 | 0.00 | 0.00 | 0.00 | 0.00 |
| Hospital 1 | 2021-08-25 | 18.33 | 2.85 | 4.09 | 0.00 | 100.00 | 0.00 |
| Hospital 2 | 2021-08-25 | 301.43 | 33.20 | 49.68 | 0.00 | 100.00 | 0.00 |
| Hospital 3A | 2021-08-25 | 6.14 | 2.05 | 2.75 | 0.00 | 100.00 | 0.00 |
| Hospital 3B | 2021-08-25 | 204.17 | 24.83 | 36.31 | 0.00 | 100.00 | 0.00 |
| Hospital 3C | 2021-08-25 | 11.43 | 0.00 | 0.00 | 0.00 | 0.00 | 0.00 |
| Hospital 1 | 2021-08-30 | 19.15 | 5.45 | 7.21 | 0.00 | 100.00 | 0.00 |
| Hospital 2 | 2021-08-30 | 2894.39 | 526.51 | 889.96 | 0.00 | 100.00 | 0.00 |
| Hospital 3A | 2021-08-30 | 3.86 | 3.70 | 4.93 | 0.00 | 100.00 | 0.00 |
| Hospital 3B | 2021-08-30 | 3.38 | 3.84 | 5.12 | 0.00 | 100.00 | 0.00 |
| Hospital 3C | 2021-08-30 | 287.38 | 44.64 | 68.88 | 0.00 | 100.00 | 0.00 |
| Hospital 1 | 2021-09-01 | 108.53 | 21.44 | 43.84 | 0.00 | 100.00 | 0.00 |
| Hospital 2 | 2021-09-01 | 20.45 | 1.98 | 4.03 | 0.00 | 100.00 | 0.00 |
| Hospital 3A | 2021-09-01 | 37.59 | 24.64 | 49.80 | 0.00 | 100.00 | 0.00 |
| Hospital 3B | 2021-09-01 | 46.16 | 7.74 | 16.05 | 0.00 | 100.00 | 0.00 |
| Hospital 2 | 2021-09-06 | 33.82 | 7.91 | 15.43 | 0.00 | 100.00 | 0.00 |
| Hospital 2 | 2021-09-08 | 134.17 | 22.41 | 44.29 | 0.00 | 100.00 | 0.00 |
| Hospital 1 | 2021-09-13 | 1280.14 | 120.34 | 249.16 | 0.00 | 100.00 | 0.00 |
| Hospital 2 | 2021-09-13 | 220.12 | 26.27 | 51.45 | 0.00 | 100.00 | 0.00 |
| Hospital 3A | 2021-09-13 | 209.17 | 47.34 | 94.81 | 0.00 | 100.00 | 0.00 |
| Hospital 3B | 2021-09-13 | 67.52 | 2.29 | 4.17 | 0.00 | 100.00 | 0.00 |
| Hospital 3C | 2021-09-13 | 56.97 | 3.56 | 6.71 | 0.00 | 100.00 | 0.00 |
| Hospital 2 | 2021-09-15 | 486.45 | 72.87 | 146.98 | 0.00 | 100.00 | 0.00 |
| Hospital 3A | 2021-09-15 | 73.54 | 31.36 | 64.79 | 0.00 | 100.00 | 0.00 |
| Hospital 3B | 2021-09-15 | 1.92 | 0.00 | 0.00 | 0.00 | 0.00 | 0.00 |
| Hospital 3C | 2021-09-15 | 1.31 | 0.00 | 0.00 | 0.00 | 0.00 | 0.00 |
| Hospital 1 | 2021-09-20 | 90.77 | 13.60 | 25.39 | 0.00 | 100.00 | 0.00 |
| Hospital 2 | 2021-09-20 | 178.45 | 54.80 | 106.79 | 0.00 | 100.00 | 0.00 |
| Hospital 3A | 2021-09-20 | 5.39 | 2.52 | 4.37 | 0.00 | 100.00 | 0.00 |
| Hospital 3B | 2021-09-20 | 23.95 | 4.30 | 7.85 | 0.00 | 100.00 | 0.00 |
| Hospital 1 | 2021-09-22 | 389.21 | 8.30 | 15.60 | 0.00 | 100.00 | 0.00 |
| Hospital 2 | 2021-09-22 | 4.48 | 3.25 | 5.98 | 0.00 | 100.00 | 0.00 |
| Hospital 3A | 2021-09-22 | 1.41 | 1.93 | 3.64 | 0.00 | 100.00 | 0.00 |
| Hospital 3B | 2021-09-22 | 0.98 | 0.00 | 0.00 | 0.00 | 0.00 | 0.00 |
| Hospital 3C | 2021-09-22 | 2.25 | 1.36 | 2.58 | 0.00 | 100.00 | 0.00 |
| Hospital 2 | 2021-09-27 | 13.53 | 1.97 | 3.56 | 0.00 | 100.00 | 0.00 |
| Hospital 3A | 2021-09-27 | 2.00 | 2.85 | 5.22 | 0.00 | 100.00 | 0.00 |
| Hospital 3B | 2021-09-27 | 5.11 | 2.47 | 4.51 | 0.00 | 100.00 | 0.00 |
| Hospital 1 | 2021-09-29 | 156.05 | 17.05 | 31.91 | 0.00 | 100.00 | 0.00 |
| Hospital 2 | 2021-09-29 | 73.47 | 9.69 | 18.36 | 0.00 | 100.00 | 0.00 |
| Hospital 3A | 2021-09-29 | 20.15 | 3.17 | 5.79 | 0.00 | 100.00 | 0.00 |
| Hospital 3B | 2021-09-29 | 9.42 | 0.70 | 0.70 | 0.00 | 100.00 | 0.00 |
| Hospital 3C | 2021-09-29 | 4.40 | 0.94 | 1.72 | 0.00 | 100.00 | 0.00 |
| Hospital 1 | 2021-10-04 | 133.82 | 15.73 | 29.68 | 0.00 | 100.00 | 0.00 |
| Hospital 2 | 2021-10-04 | 8.21 | 0.66 | 0.19 | 0.00 | 100.00 | 0.00 |
| Hospital 3A | 2021-10-04 | 19.89 | 4.99 | 9.59 | 0.00 | 100.00 | 0.00 |
| Hospital 3B | 2021-10-04 | 3.05 | 0.00 | 0.00 | 0.00 | 0.00 | 0.00 |
| Hospital 1 | 2021-10-06 | 54.04 | 25.85 | 49.34 | 0.00 | 100.00 | 0.00 |
| Hospital 2 | 2021-10-06 | 391.57 | 98.46 | 202.16 | 0.00 | 100.00 | 0.00 |
| Hospital 3A | 2021-10-06 | 17.21 | 10.67 | 20.38 | 0.00 | 100.00 | 0.00 |
| Hospital 3B | 2021-10-06 | 0.81 | 0.41 | 0.70 | 0.00 | 100.00 | 0.00 |
| Hospital 3C | 2021-10-06 | 0.39 | 0.41 | 0.71 | 0.00 | 100.00 | 0.00 |
| Hospital 1 | 2021-10-13 | 45.53 | 12.01 | 21.46 | 0.00 | 100.00 | 0.00 |
| Hospital 2 | 2021-10-13 | 102.41 | 20.69 | 39.19 | 0.00 | 100.00 | 0.00 |
| Hospital 3A | 2021-10-13 | 32.37 | 6.67 | 11.90 | 0.00 | 100.00 | 0.00 |
| Hospital 3B | 2021-10-13 | 18.82 | 9.38 | 17.57 | 0.00 | 100.00 | 0.00 |
| Hospital 3C | 2021-10-13 | 2.32 | 0.00 | 0.00 | 0.00 | 0.00 | 0.00 |
| Hospital 1 | 2021-10-18 | 162.59 | 13.46 | 25.80 | 0.00 | 100.00 | 0.00 |
| Hospital 2 | 2021-10-18 | 5.84 | 0.78 | 1.36 | 0.00 | 100.00 | 0.00 |
| Hospital 3A | 2021-10-18 | 3.94 | 0.00 | 0.00 | 0.00 | 0.00 | 0.00 |
| Hospital 3B | 2021-10-18 | 10.77 | 1.26 | 2.22 | 0.00 | 100.00 | 0.00 |
| Hospital 3C | 2021-10-18 | 24.44 | 1.10 | 1.97 | 0.00 | 100.00 | 0.00 |
| Hospital 1 | 2021-10-20 | 90.22 | 11.55 | 21.36 | 0.00 | 100.00 | 0.00 |
| Hospital 2 | 2021-10-20 | 11.14 | 5.28 | 9.44 | 0.00 | 100.00 | 0.00 |
| Hospital 3B | 2021-10-20 | 2.15 | 0.00 | 0.00 | 0.00 | 0.00 | 0.00 |
| Hospital 1 | 2021-10-25 | 5.87 | 0.00 | 0.00 | 0.00 | 0.00 | 0.00 |
| Hospital 2 | 2021-10-25 | 26.71 | 5.42 | 10.01 | 0.00 | 100.00 | 0.00 |
| Hospital 3A | 2021-10-25 | 14.60 | 2.93 | 5.29 | 0.00 | 100.00 | 0.00 |
| Hospital 3B | 2021-10-25 | 2.77 | 0.00 | 0.00 | 0.00 | 0.00 | 0.00 |
| Hospital 1 | 2021-10-27 | 191.86 | 57.59 | 112.12 | 0.00 | 100.00 | 0.00 |
| Hospital 2 | 2021-10-27 | 3.76 | 0.76 | 3.05 | 0.00 | 100.00 | 0.00 |
| Hospital 3B | 2021-10-27 | 120.86 | 44.52 | 112.46 | 0.00 | 100.00 | 0.00 |
| Hospital 1 | 2021-11-01 | 63.67 | 15.98 | 34.21 | 0.00 | 100.00 | 0.00 |
| Hospital 2 | 2021-11-01 | 136.82 | 25.10 | 56.98 | 0.00 | 100.00 | 0.00 |
| Hospital 3B | 2021-11-01 | 9.78 | 1.37 | 2.31 | 0.00 | 100.00 | 0.00 |
| Hospital 1 | 2021-11-03 | 10.04 | 2.37 | 4.26 | 0.00 | 100.00 | 0.00 |
| Hospital 2 | 2021-11-03 | 5.91 | 0.00 | 0.00 | 0.00 | 0.00 | 0.00 |
| Hospital 1 | 2021-11-08 | 6.37 | 0.62 | 1.03 | 0.00 | 100.00 | 0.00 |
| Hospital 3A | 2021-11-08 | 58.53 | 35.50 | 80.68 | 0.00 | 100.00 | 0.00 |
| Hospital 1 | 2021-11-10 | 3.48 | 0.00 | 0.00 | 0.00 | 0.00 | 0.00 |
| Hospital 2 | 2021-11-10 | 0.73 | 0.00 | 0.00 | 0.00 | 0.00 | 0.00 |
| Hospital 3A | 2021-11-10 | 33.41 | 53.43 | 133.59 | 0.00 | 100.00 | 0.00 |
| Hospital 3B | 2021-11-10 | 1.53 | 0.74 | 1.23 | 0.00 | 100.00 | 0.00 |
| Hospital 1 | 2021-11-15 | 10.89 | 0.71 | 4.08 | 0.00 | 100.00 | 0.00 |
| Hospital 3A | 2021-11-15 | 50.82 | 33.28 | 76.88 | 0.00 | 100.00 | 0.00 |
| Hospital 3B | 2021-11-15 | 35.91 | 14.68 | 30.57 | 0.00 | 100.00 | 0.00 |
| Hospital 1 | 2021-11-17 | 3.43 | 3.36 | 6.00 | 0.00 | 100.00 | 0.00 |
| Hospital 2 | 2021-11-17 | 9.92 | 2.45 | 4.15 | 0.00 | 100.00 | 0.00 |
| Hospital 3A | 2021-11-17 | 2.31 | 0.00 | 0.00 | 0.00 | 0.00 | 0.00 |
| Hospital 3C | 2021-11-17 | 14.15 | 5.79 | 11.19 | 0.00 | 100.00 | 0.00 |
| Hospital 1 | 2021-11-22 | 10.66 | 6.42 | 12.23 | 0.00 | 100.00 | 0.00 |
| Hospital 3A | 2021-11-22 | 6.93 | 4.73 | 8.81 | 0.00 | 100.00 | 0.00 |
| Hospital 1 | 2021-11-24 | 1.66 | 1.88 | 3.45 | 0.00 | 100.00 | 0.00 |
| Hospital 2 | 2021-11-24 | 5.96 | 15.92 | 33.04 | 0.00 | 100.00 | 0.00 |
| Hospital 3A | 2021-11-24 | 206.26 | 172.89 | 504.12 | 0.00 | 100.00 | 0.00 |
| Hospital 1 | 2021-11-29 | 30.73 | 12.68 | 27.93 | 0.00 | 100.00 | 0.00 |
| Hospital 3A | 2021-11-29 | 8.18 | 8.80 | 18.19 | 0.00 | 100.00 | 0.00 |
| Hospital 1 | 2021-12-01 | 27.06 | 1.75 | 2.96 | 0.00 | 100.00 | 0.00 |
| Hospital 3A | 2021-12-01 | 2.91 | 0.00 | 0.00 | 0.00 | 0.00 | 0.00 |
| Hospital 1 | 2021-12-06 | 54.89 | 10.57 | 20.75 | 0.00 | 100.00 | 0.00 |
| Hospital 3A | 2021-12-06 | 20.01 | 0.00 | 0.54 | 0.00 | 100.00 | 0.00 |
| Hospital 1 | 2021-12-08 | 35.15 | 3.62 | 6.19 | 0.00 | 100.00 | 0.00 |
| Hospital 1 | 2021-12-13 | 26.06 | 4.67 | 8.17 | 0.00 | 100.00 | 0.00 |
| Hospital 3A | 2021-12-13 | 36.44 | 36.75 | 85.05 | 0.00 | 100.00 | 0.00 |
| Hospital 3C | 2021-12-13 | 6.54 | 3.96 | 7.09 | 0.00 | 100.00 | 0.00 |
| Hospital 1 | 2021-12-15 | 161.55 | 62.51 | 154.82 | 0.00 | 100.00 | 0.00 |
| Hospital 3A | 2021-12-15 | 292.66 | 57.47 | 144.48 | 0.00 | 100.00 | 0.00 |
| Hospital 3C | 2021-12-15 | 2.30 | 1.14 | 0.00 | 1.00 | 0.00 | 100.00 |
| Hospital 1 | 2021-12-20 | 427.56 | 148.08 | 393.22 | 0.00 | 100.00 | 0.00 |
| Hospital 2 | 2021-12-20 | 45.37 | 11.88 | 9.29 | 5.78 | 61.63 | 38.37 |
| Hospital 3A | 2021-12-20 | 129.17 | 64.00 | 151.40 | 0.00 | 100.00 | 0.00 |
| Hospital 3C | 2021-12-20 | 11.10 | 11.92 | 0.00 | 9.49 | 0.00 | 100.00 |
| Hospital 2 | 2021-12-29 | 40.71 | 28.77 | 17.70 | 16.26 | 52.12 | 47.88 |
| Hospital 1 | 2021-12-30 | 1329.59 | 367.02 | 191.03 | 273.21 | 41.15 | 58.85 |
| Hospital 3A | 2021-12-30 | 118.40 | 47.80 | 32.70 | 30.22 | 51.97 | 48.03 |
| Hospital 3B | 2021-12-30 | 18.95 | 1.36 | 0.00 | 0.98 | 0.00 | 100.00 |
| Hospital 3C | 2021-12-30 | 17.58 | 16.23 | 0.00 | 12.54 | 0.00 | 100.00 |
| Hospital 1 | 2022-01-03 | 294.99 | 273.32 | 0.00 | 238.84 | 0.00 | 100.00 |
| Hospital 3A | 2022-01-03 | 303.19 | 361.01 | 0.28 | 326.11 | 0.09 | 99.91 |
| Hospital 3B | 2022-01-03 | 20.89 | 12.56 | 0.00 | 8.97 | 0.00 | 100.00 |
| Hospital 3C | 2022-01-03 | 86.02 | 149.22 | 11.57 | 117.80 | 8.94 | 91.06 |
| Hospital 1 | 2022-01-05 | 957.26 | 363.61 | 53.96 | 307.53 | 14.93 | 85.07 |
| Hospital 2 | 2022-01-05 | 167.81 | 177.68 | 0.00 | 161.45 | 0.00 | 100.00 |
| Hospital 3A | 2022-01-05 | 323.39 | 296.94 | 0.00 | 272.31 | 0.00 | 100.00 |
| Hospital 3B | 2022-01-05 | 114.63 | 91.77 | 0.00 | 82.29 | 0.00 | 100.00 |
| Hospital 1 | 2022-01-10 | 878.28 | 395.23 | 0.00 | 382.85 | 0.00 | 100.00 |
| Hospital 2 | 2022-01-10 | 883.13 | 230.79 | 0.00 | 217.70 | 0.00 | 100.00 |
| Hospital 3A | 2022-01-10 | 88.72 | 82.12 | 0.00 | 70.19 | 0.00 | 100.00 |
| Hospital 3B | 2022-01-10 | 338.02 | 106.31 | 0.00 | 80.99 | 0.00 | 100.00 |
| Hospital 3C | 2022-01-10 | 165.23 | 54.68 | 4.22 | 40.48 | 9.44 | 90.56 |
| Hospital 1 | 2022-01-12 | 1129.54 | 215.94 | 0.00 | 177.94 | 0.00 | 100.00 |
| Hospital 3A | 2022-01-12 | 20.82 | 24.72 | 0.00 | 20.39 | 0.00 | 100.00 |
| Hospital 3B | 2022-01-12 | 2166.06 | 348.51 | 0.00 | 298.59 | 0.00 | 100.00 |
| Hospital 3C | 2022-01-12 | 46.26 | 44.18 | 0.00 | 38.51 | 0.00 | 100.00 |
| Hospital 1 | 2022-01-13 | 1991.08 | 969.81 | 0.00 | 900.85 | 0.00 | 100.00 |
| Hospital 3A | 2022-01-13 | 45.83 | 22.10 | 0.00 | 19.38 | 0.00 | 100.00 |
| Hospital 3B | 2022-01-13 | 15621.47 | 4875.84 | 0.00 | 4731.22 | 0.00 | 100.00 |
| Hospital 3C | 2022-01-13 | 68.03 | 57.51 | 0.00 | 55.53 | 0.00 | 100.00 |
| Hospital 1 | 2022-01-17 | 622.56 | 477.72 | 0.00 | 466.06 | 0.00 | 100.00 |
| Hospital 2 | 2022-01-17 | 246.92 | 253.75 | 0.00 | 247.65 | 0.00 | 100.00 |
| Hospital 3A | 2022-01-17 | 40.14 | 34.64 | 0.00 | 30.95 | 0.00 | 100.00 |
| Hospital 3B | 2022-01-17 | 60.22 | 19.16 | 0.00 | 15.61 | 0.00 | 100.00 |
| Hospital 3C | 2022-01-17 | 10.35 | 13.88 | 0.00 | 11.09 | 0.00 | 100.00 |
| Hospital 1 | 2022-01-19 | 805.04 | 329.79 | 0.00 | 290.95 | 0.00 | 100.00 |
| Hospital 2 | 2022-01-19 | 818.97 | 337.09 | 0.00 | 290.54 | 0.00 | 100.00 |
| Hospital 3A | 2022-01-19 | 38.28 | 33.25 | 0.00 | 30.48 | 0.00 | 100.00 |
| Hospital 3B | 2022-01-19 | 839.79 | 161.31 | 0.00 | 158.54 | 0.00 | 100.00 |
| Hospital 3C | 2022-01-19 | 264.67 | 243.19 | 0.00 | 236.63 | 0.00 | 100.00 |
| Hospital 1 | 2022-01-20 | 517.23 | 436.06 | 0.00 | 428.47 | 0.00 | 100.00 |
| Hospital 2 | 2022-01-20 | 214.19 | 216.63 | 0.00 | 224.53 | 0.00 | 100.00 |
| Hospital 3A | 2022-01-20 | 58.92 | 67.36 | 0.00 | 64.43 | 0.00 | 100.00 |
| Hospital 3B | 2022-01-20 | 95.04 | 85.84 | 0.00 | 79.79 | 0.00 | 100.00 |
| Hospital 3C | 2022-01-20 | 128.64 | 174.41 | 0.00 | 161.68 | 0.00 | 100.00 |
| Hospital 1 | 2022-01-24 | 696.18 | 444.95 | 0.00 | 398.82 | 0.00 | 100.00 |
| Hospital 2 | 2022-01-24 | 57.89 | 36.67 | 0.00 | 32.97 | 0.00 | 100.00 |
| Hospital 3A | 2022-01-24 | 27.13 | 59.75 | 0.00 | 57.01 | 0.00 | 100.00 |
| Hospital 3C | 2022-01-24 | 283.49 | 348.12 | 0.00 | 328.24 | 0.00 | 100.00 |
| Hospital 1 | 2022-01-26 | 1329.43 | 618.85 | 0.00 | 594.64 | 0.00 | 100.00 |
| Hospital 2 | 2022-01-26 | 310.15 | 145.23 | 0.00 | 144.14 | 0.00 | 100.00 |
| Hospital 3A | 2022-01-26 | 27.20 | 27.42 | 0.00 | 29.11 | 0.00 | 100.00 |
| Hospital 3B | 2022-01-26 | 1040.15 | 196.99 | 0.00 | 191.09 | 0.00 | 100.00 |
| Hospital 3C | 2022-01-26 | 160.94 | 125.60 | 0.00 | 128.88 | 0.00 | 100.00 |
| Hospital 1 | 2022-01-27 | 741.80 | 477.00 | 0.00 | 488.79 | 0.00 | 100.00 |
| Hospital 2 | 2022-01-27 | 1496.45 | 504.42 | 0.00 | 509.87 | 0.00 | 100.00 |
| Hospital 3A | 2022-01-27 | 17.34 | 14.50 | 0.00 | 12.46 | 0.00 | 100.00 |
| Hospital 3B | 2022-01-27 | 365.84 | 149.96 | 0.00 | 138.29 | 0.00 | 100.00 |
| Hospital 3C | 2022-01-27 | 1611.74 | 1043.43 | 0.00 | 1010.69 | 0.00 | 100.00 |
| Hospital 1 | 2022-01-31 | 1175.93 | 806.10 | 0.00 | 792.67 | 0.00 | 100.00 |
| Hospital 2 | 2022-01-31 | 166.47 | 169.47 | 0.00 | 156.95 | 0.00 | 100.00 |
| Hospital 3A | 2022-01-31 | 30.62 | 25.37 | 0.00 | 24.26 | 0.00 | 100.00 |
| Hospital 3B | 2022-01-31 | 474.17 | 222.34 | 0.00 | 220.45 | 0.00 | 100.00 |
| Hospital 3C | 2022-01-31 | 361.82 | 311.80 | 0.00 | 307.92 | 0.00 | 100.00 |

**SUPPLEMENTARY FIGURES**

**
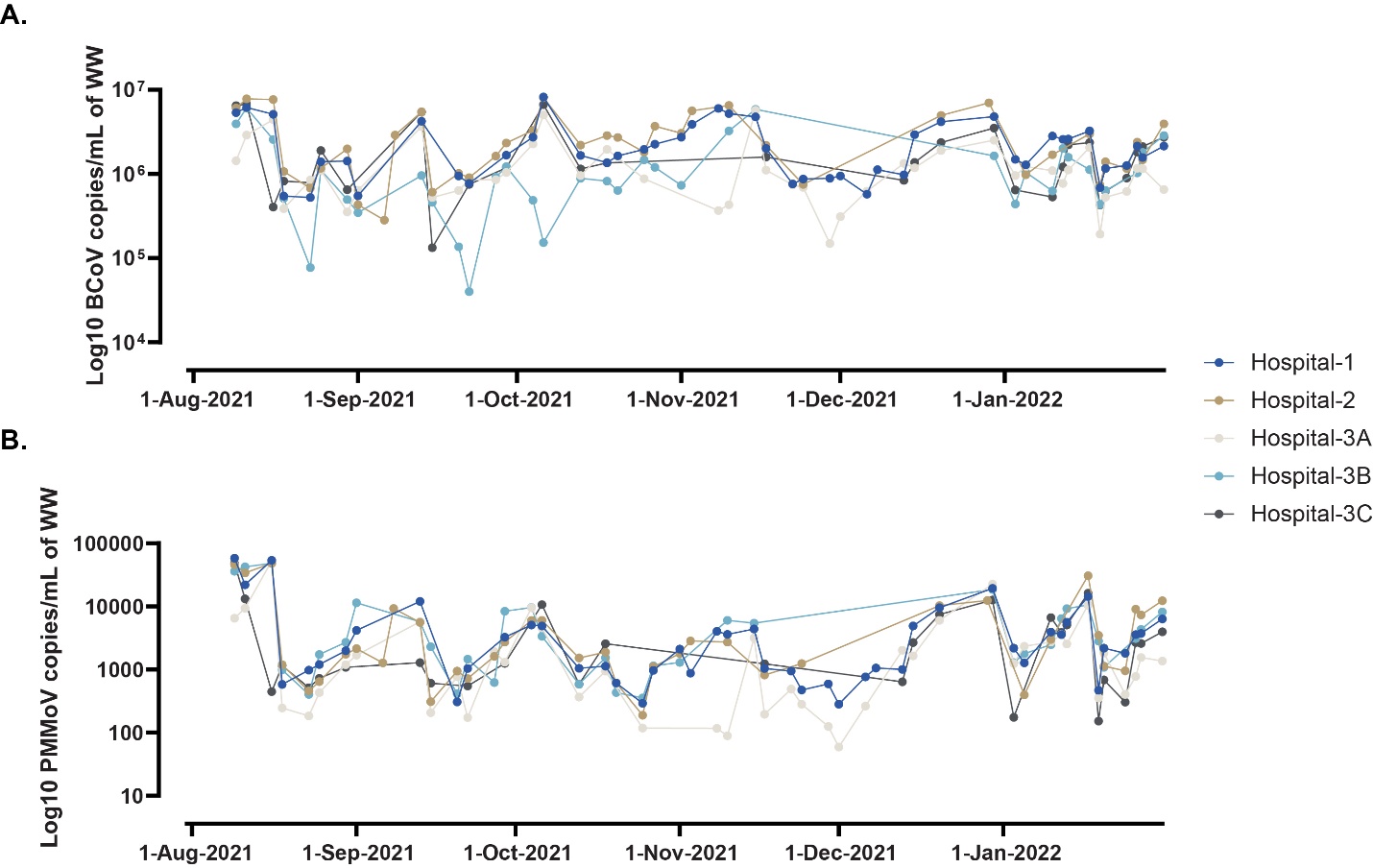
**

**Figure 1S. Quantification of controls in wastewater hospital samples.** Genomic copies per ml of wastewater of **A)** Bovine coronavirus (BCoV) or **B)** Pepper mild mottle virus (PMMoV) processed from hospital locations over time. The figure shows the average of three technical replicates in the Log10 scale.

**
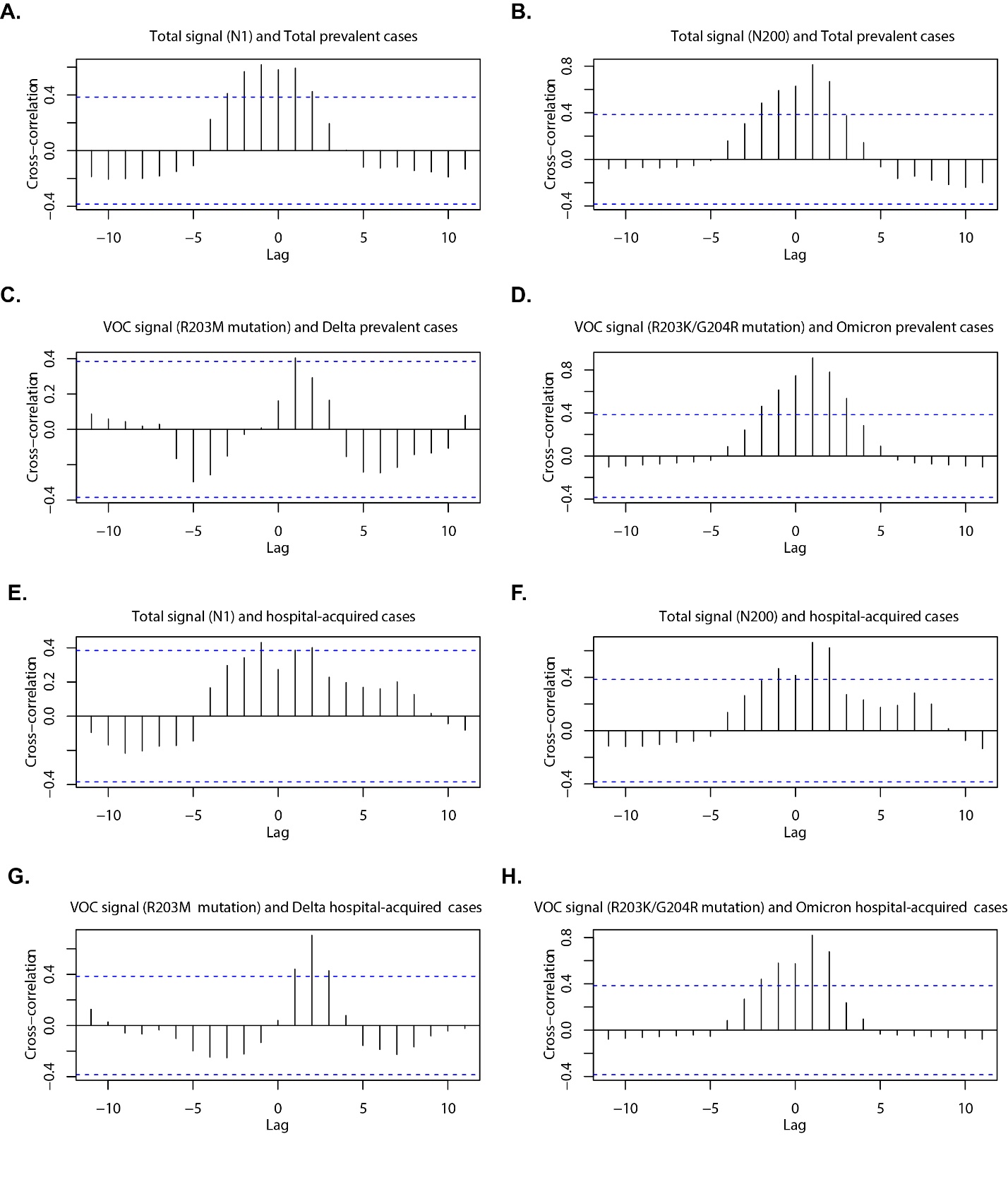
**

**Figure 2S. Cross-correlation function (CCF) of wastewater data and clinical data for Hospital-1.** Figures A to D show the CCF analysis between prevalent cases (Total or VOC-specific) and wastewater signal measured with either N1 (**A**), N200 (**B**), R203M (**C**) or R203K/G204R (**D**) signals. Figures E-H show the CCF analysis between hospital-acquired cases (Total or VOC-specific) and wastewater signal measured with either N1 (**E**), N200 (**F**), R203M (**G**) or R203K/G204R (**H**) signals. Blue dashed lines indicate confidence threshold for α = 0.05.

**
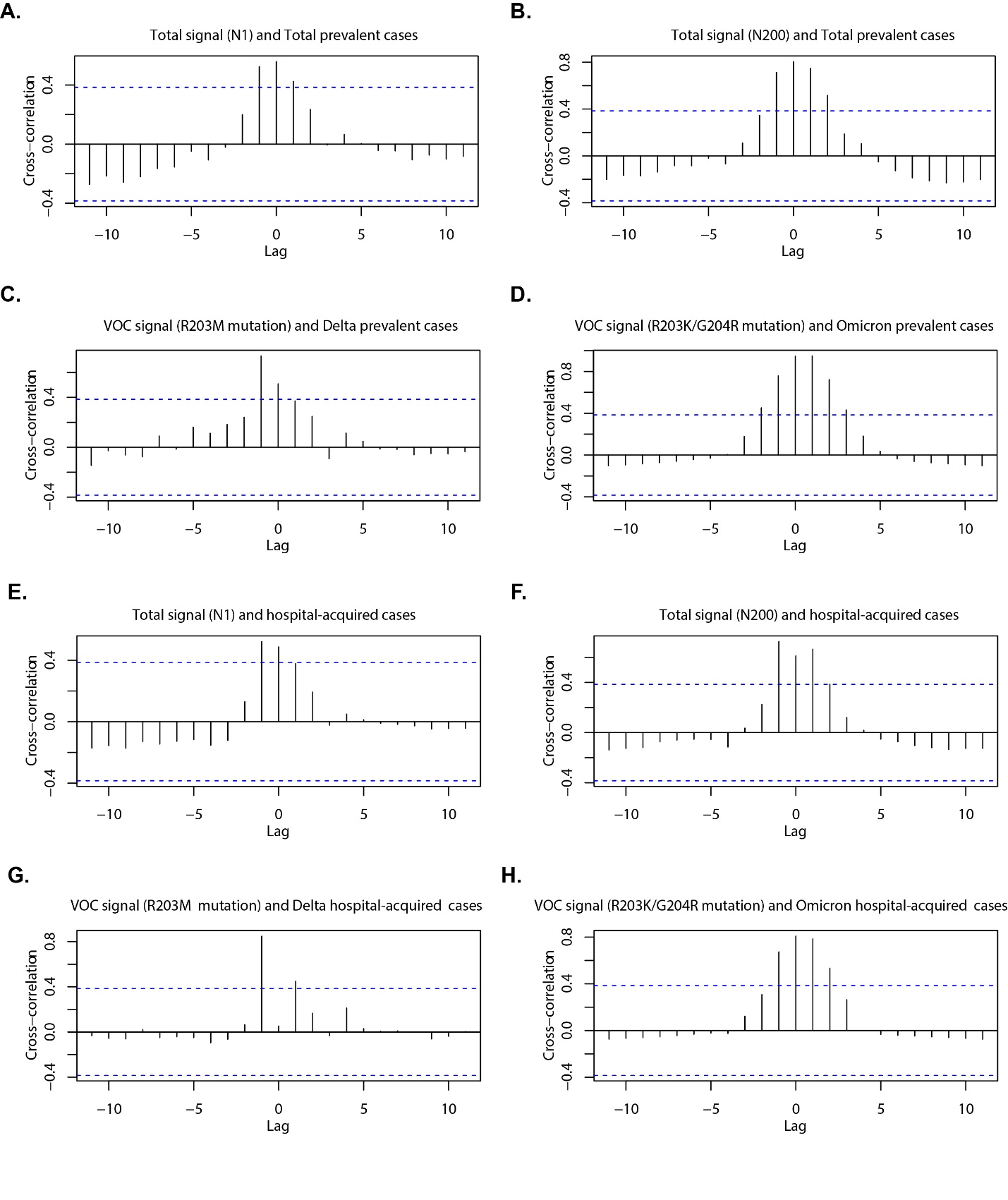
**

**Figure 3S. Cross-correlation function (CCF) of wastewater data and clinical data at Hospital-2.** Figures A to D show the CCF analysis between prevalent cases (Total or VOC-specific) and wastewater signal measured with either N1 (**A**), N200 (**B**), R203M (**C**) or R203K/G204R (**D**) signals. Figures E-H show the CCF analysis between hospital-acquired cases (Total or VOC-specific) and wastewater signal measured with either N1 (**E**), N200 (**F**), R203M (**G**) or R203K/G204R (**H**) signals. Blue dashed lines indicate confidence threshold for α = 0.05.

**
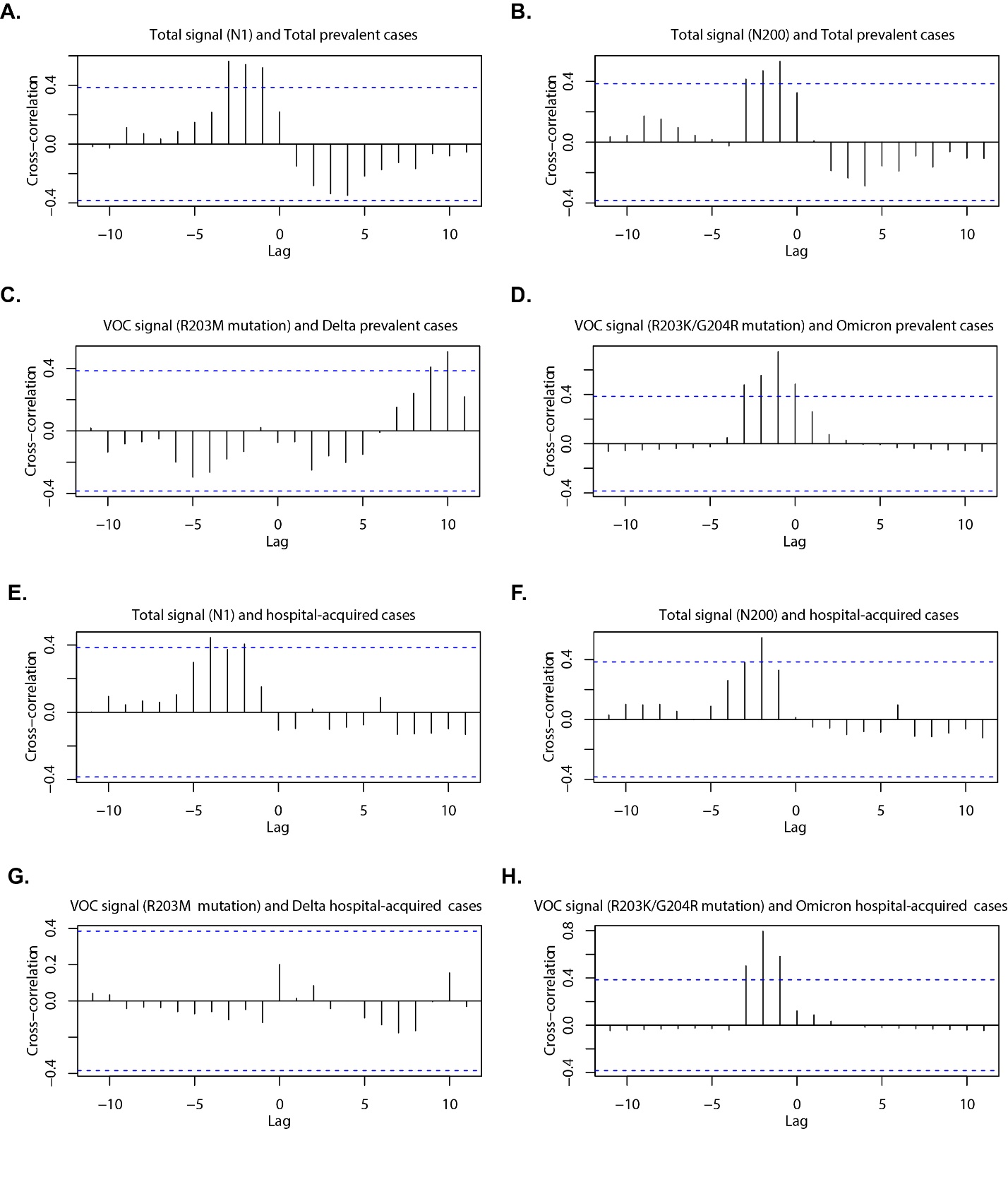
**

**Figure 4S. Cross-correlation function (CCF) of wastewater data and clinical data at Hospital-3 sub-location A.** Figures A to D show the CCF analysis between prevalent cases (Total or VOC-specific) and wastewater signal measured with either N1 (**A**), N200 (**B**), R203M (**C**) or R203K/G204R (**D**) signals. Figures E-H show the CCF analysis between hospital-acquired cases (Total or VOC-specific) and wastewater signal measured with either N1 (**E**), N200 (**F**), R203M (**G**) or R203K/G204R (**H**) signals. Blue dashed lines indicate confidence threshold for α = 0.05.

**
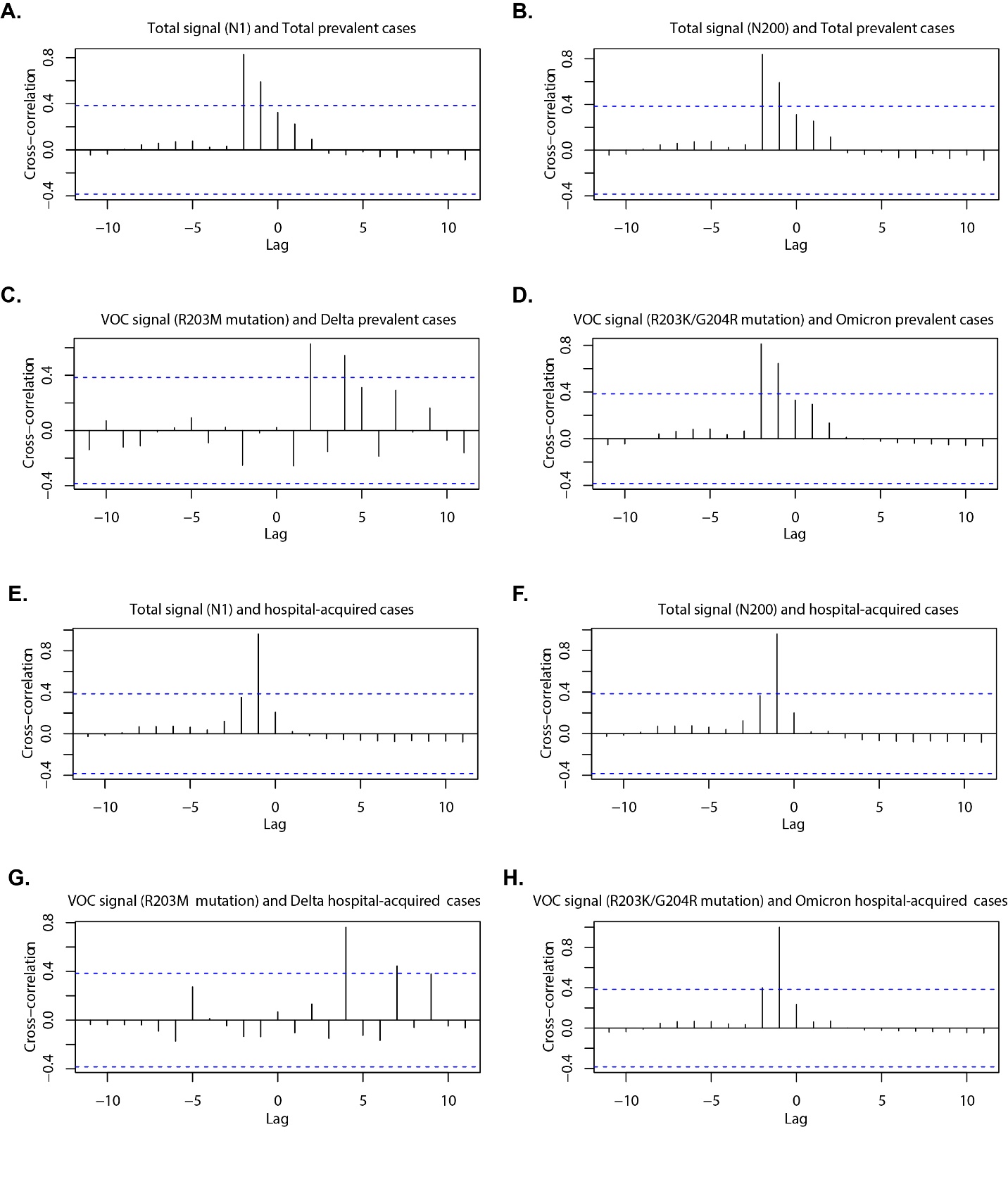
**

**Figure 5S. Cross-correlation function (CCF) of wastewater data and clinical data at Hospital-3 sub-location B.** Figures A to D show the CCF analysis between prevalent cases (Total or VOC-specific) and wastewater signal measured with either N1 (**A**), N200 (**B**), R203M (**C**) or R203K/G204R (**D**) signals. Figures E-H show the CCF analysis between hospital-acquired cases (Total or VOC-specific) and wastewater signal measured with either N1 (**E**), N200 (**F**), R203M (**G**) or R203K/G204R (**H**) signals. Blue dashed lines indicate confidence threshold for α = 0.05.

**
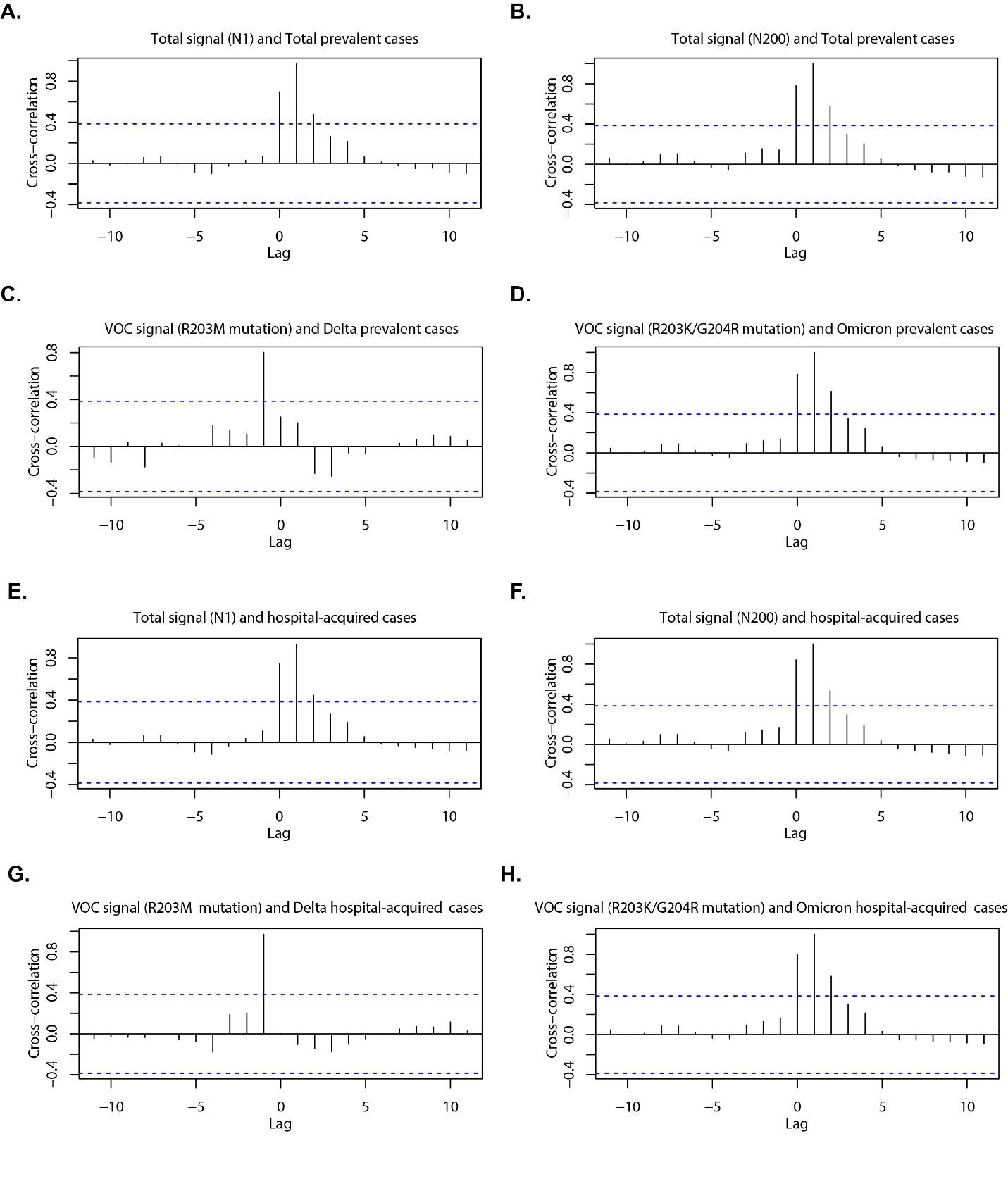
**

**Figure 6S. Cross-correlation function (CCF) of wastewater data and clinical data at Hospital-3 sub-location C.** Figures A to D show the CCF analysis between prevalent cases (Total or VOC-specific) and wastewater signal measured with either N1 (**A**), N200 (**B**), R203M (**C**) or R203K/G204R (**D**) signals. Figures E-H show the CCF analysis between hospital-acquired cases (Total or VOC-specific) and wastewater signal measured with either N1 (**E**), N200 (**F**), R203M (**G**) or R203K/G204R (**H**) signals. Blue dashed lines indicate confidence threshold for α = 0.05.

**
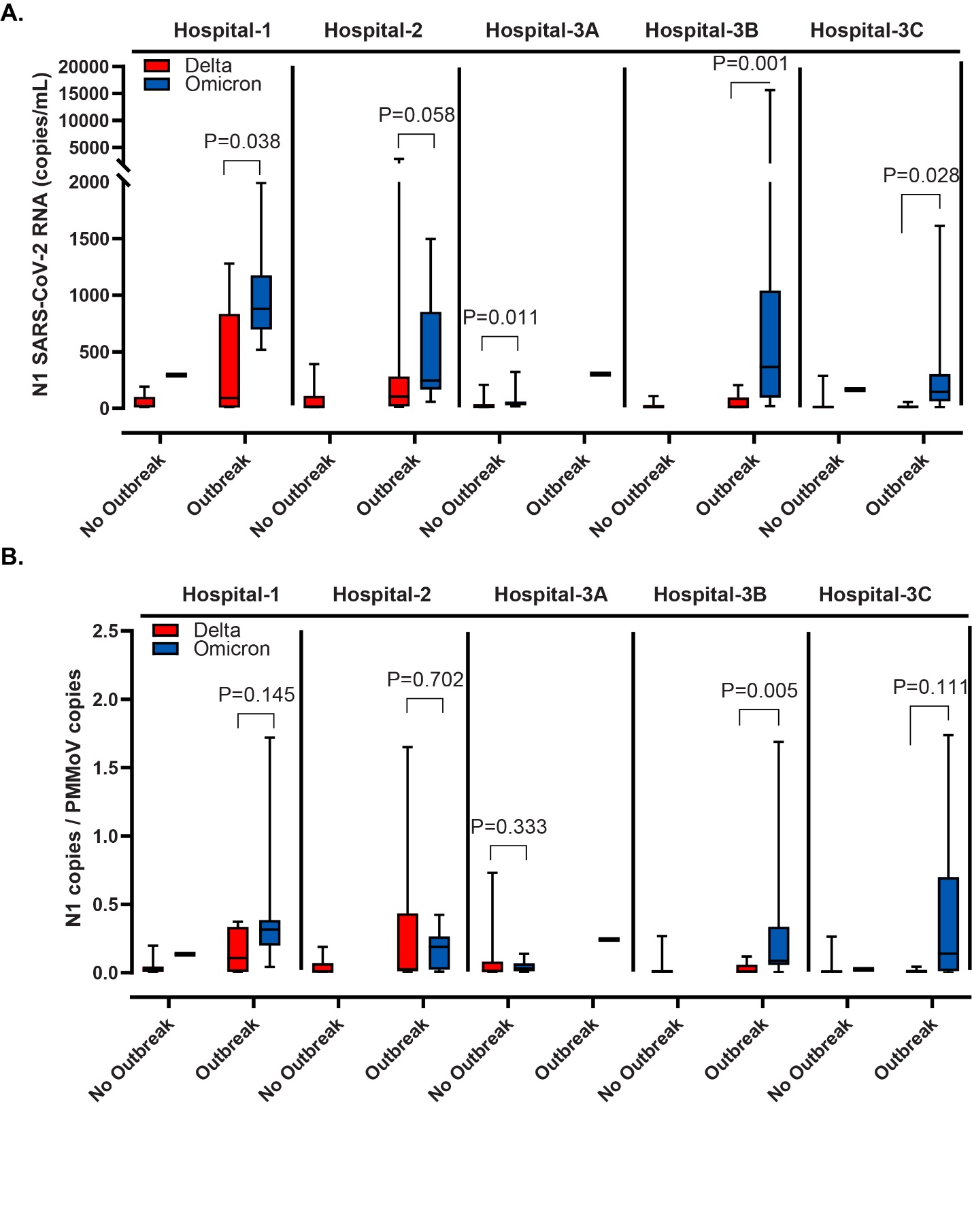
**

**Figure 7S. Abundance of SARS-CoV-2 wastewater signal during Delta or Omicron as a function of outbreak status.** SARS-CoV-2 RNA data from the Delta-wave (i.e., mid-August to end of November 2021) or Omicron-wave (i.e., January 2022) were compared from samples collected during outbreak-free periods or within 5 days of an outbreak being declared. **A)** N1 SARS-CoV-2 RNA signal (copies/mL) and **B)** N1 SARS-CoV-2 genomic copies normalized to genomic copies of the fecal biomarker PMMoV. Median and interquartile ranges are indicated as the middle, top, and bottom lines of each box. Ends of the whiskers mark the lowest and highest signal determined in each category for each hospital analyzed. Differences were determined using the Mann Whitney U test.

**REFERENCES**

1. Acosta N, Bautista MA, Hollman J, et al. A Multicenter Study Investigating SARS-CoV-2 in Tertiary-Care Hospital Wastewater. Viral burden correlates with increasing hospitalized cases as well as hospital-associated transmissions and outbreaks. *Water Research* 2021: 117369.

2. Whitney ON, Kennedy LC, Fan VB, et al. Sewage, Salt, Silica, and SARS-CoV-2 (4S): An Economical Kit-Free Method for Direct Capture of SARS-CoV-2 RNA from Wastewater. *Environmental Science & Technology* 2021; 55(8): 4880-8.

3. Hubert CRJ, Acosta N, Waddell BJM, et al. Tracking Emergence and Spread of SARS-CoV-2 Omicron Variant in Large and Small Communities by Wastewater Monitoring in Alberta, Canada. Emerging Infectious Disease journal 2022; 28(9).

4. Pabbaraju K, Zelyas N, Wong A, et al. Evolving strategy for an evolving virus: Development of real-time PCR assays for detecting all SARS-CoV-2 variants of concern. *Journal of Virological Methods* 2022; 307: 114553.

5. Pabbaraju K, Wong AA, Douesnard M, et al. Development and validation of RT-PCR assays for testing for SARS-CoV-2. *Official Journal of the Association of Medical Microbiology and Infectious Disease Canada* 2021; 6(1): 16-22.
